# Emerging Signatures of Hematological Malignancies from Gene Expression and Transcription Factor-Gene Regulations

**DOI:** 10.3390/ijms252413588

**Published:** 2024-12-19

**Authors:** Daniele Dall’Olio, Federico Magnani, Francesco Casadei, Tommaso Matteuzzi, Nico Curti, Alessandra Merlotti, Giorgia Simonetti, Matteo Giovanni Della Porta, Daniel Remondini, Martina Tarozzi, Gastone Castellani

**Affiliations:** 1 Department of Medical and Surgical Sciences, University of Bologna, 40138 Bologna, Italy; 2 IRCCS Istituto delle Scienze Neurologiche di Bologna, 40139 Bologna, Italy; 3 Department of Physics and Astronomy, University of Firenze, 50019 Sesto Fiorentino, Italy; 4 Department of Physics and Astronomy, University of Bologna, 40127 Bologna, Italy; 5 Biosciences Laboratory, IRCCS Istituto Romagnolo per lo Studio dei Tumori (IRST) “Dino Amadori”, 47014 Meldola, Italy; 6 Comprehensive Cancer Center, IRCCS Humanitas Clinical and Research Center and Department of Biomedical Sciences, Humanitas University, 20089 Milan, Italy; 7 IRCCS Azienda Ospedaliero-Universitaria di Bologna, 40138 Bologna, Italy

**Keywords:** hematological cancers, gene regulatory networks, transcriptomics

## Abstract

Hematological malignancies are a diverse group of cancers developing in the peripheral blood, the bone marrow or the lymphatic system. Due to their heterogeneity, the identification of novel and advanced molecular signatures is essential for enhancing their characterization and facilitate its translation to new pharmaceutical solutions and eventually to clinical applications. In this study, we collected publicly available microarray data for more than five thousand subjects, across thirteen hematological malignancies. Using PANDA to estimate gene regulatory networks (GRNs), we performed hierarchical clustering and network analysis to explore transcription factor (TF) interactions and their implications on biological pathways. Our findings reveal distinct clustering patterns among leukemias and lymphomas, with notable differences in gene and TF expression profiles. Gene Set Enrichment Analysis (GSEA) identified 57 significantly enriched KEGG pathways, highlighting both common and unique biological processes across HMs. We also identified potential drug targets within these pathways, emphasizing the role of TFs such as *CEBPB* and *NFE2L1* in disease pathophysiology. Our comprehensive analysis enhances the understanding of the molecular landscape of HMs and suggests new avenues for targeted therapeutic strategies. These findings also motivate the adoption of regulatory networks, combined with modern biotechnological possibilities, for insightful pan-cancer exploratory studies.

## 1. Introduction

Hematological malignancies (HM) represent a substantial burden for healthcare systems [1], with a growing incidence in the last three decades. They refer to a heterogeneous group of malignancies originating from hematopoietic cells at different stages of differentiation and can be classified into three main categories depending on the affected cell type. Leukemias arise from leukocyte precursors and accumulate in the peripheral blood and/or the bone marrow. Leukemias are further classified depending on the rate of progression as acute (developing rapidly) or chronic (progressing slowly) and are based on the lineage from which they originate (myeloid or lymphoid lineage). Lymphomas are the second main category of HM: these tumors affect lymphocytes and can occur in the lymph nodes, thymus, or spleen. They are broadly categorized into Hodgkin and Non-Hodgkin lymphomas. Lastly, Multiple Myeloma (MM) affects differentiated plasma cells that proliferate and accumulate in the bone marrow. The complex pathophysiology and varied clinical manifestations of HMs represent a significant challenge. High-throughput transcriptomic technologies have revolutionized our understanding of these cancers by enabling comprehensive profiling of gene expression patterns. However, the volume and complexity of transcriptomic data require sophisticated analytical approaches to unravel the underlying biological mechanisms and identify potential therapeutic targets. In the last ten years, thanks to the growing number of publicly available microarray and RNA-seq datasets, gene expression studies have focused on determining both common and specific signatures across cancers [2,3,4]. The heterogeneous nature of HMs demands a large cohort of patients to provide enough representability for one disease, and to increase the possibility of capturing rare or unknown subtypes. Yet not many studies collect large datasets and, when possible, only the data for a single or a few HMs are available. Mostly built upon correlations between genes and gene expression profiling, transcriptomic findings suggest shared biomarkers across combinations of leukemias, myelomas and lymphomas. On top of that, pathway enrichment results can provide drug indications [5,6], thus motivating further experiments. Yet, while disease specificity can be described based on gene expression levels, individual genes cannot adequately capture the intricate and vast interplay of processes that characterize and distinguish different diseases. Conversely, recent developments on gene regulatory networks (GRNs) across tissues proved to provide an innovative perspective on gene regulation for pan-cancer studies [7,8]. Gene regulatory networks estimate the interactions between transcription factors (TFs) and their target genes, providing a powerful framework for understanding the regulatory architecture of gene expression. These networks serve as mathematical models estimating complex interactions among TFs, genes, and, when available, proteins in the form of networks. The particular interest in TFs also stems from recent discoveries about the feasibility of drug-targeting transcription factors, a strategy once deemed impossible [9]. Analyzing these networks and transcriptomic data, we aimed to uncover key regulatory genes and pathways that drive the oncogenic processes in different hematological malignancies.

This work seeks to characterize HMs based on gene expression and GRNs. While our gene expression analyses aim at those genes that are likely over-expressed for an HM, our analyses on GRNs point out interactions between TFs and genes that are likely involved for an HM. In both tasks, we look for specificities (what makes an HM unique compared to the others), and similarities (what emerges to be shared by multiple HMs). We use publicly available transcriptomic data to gather thirteen HMs: four acute and chronic leukemias, seven types of lymphoma, Myelodysplastic Syndromes (MDS), and Multiple Myeloma (MM). By exploring specificities and similarities, we also investigate the potential implications on biological pathways to gain insight into the disruption of relevant biological functions. Given the growing attention to drug repurposing as a powerful and efficient strategy to introduce novel treatments [10], we provide putative drug targets (along with their biological context) emerging from our analyses. This work contributes to shedding light on the transcriptomics landscape of HMs and motivates further laboratory experiments to perform pre-clinical validation.

## 2. Results

### 2.1. Hierarchical Clustering Based on Gene Expression

**Figure 1:**
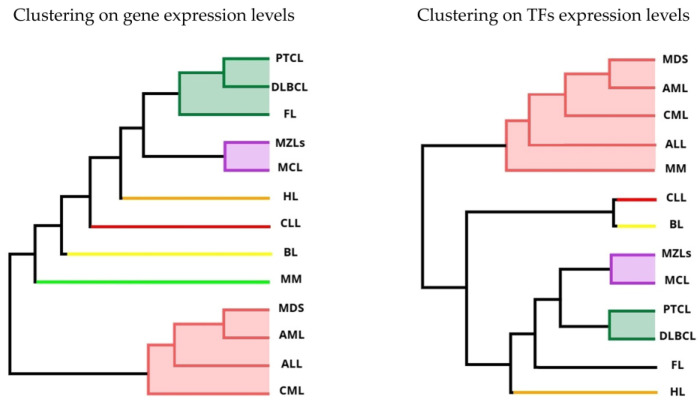
Hierarchical clustering of HMs based on a similarity score over gene expression levels (**left**) and TFs expression levels (**right**). Branches of different colors identify the optimal clustering of diseases, with respect to the aforementioned mutual similarities.

This study includes microarray data from 34 datasets (Supplementary Table S3) covering thirteen HMs: Acute Lymphocytic Leukemia (ALL), Acute Myeloid Leukemia (AML), Chronic Lymphocytic Leukemia (CLL), Chronic Myeloid Leukemia (CML), Burkitt Lymphoma (BL), Diffuse Large B-Cell Lymphoma (DLBCL), Follicular Lymphoma (FL), Hodgkin Lymphoma (HL), Mantle Cell Lymphoma (MCL), Marginal Zone Lymphomas (MZLs), Peripheral T-Cell Lymphoma (PTCL), MDS and MM. We categorize all HMs using hierarchical clustering based separately on their overall gene expression levels and TFs expression levels. Dendograms shown in Figure 1 highlight the HMs with similar expression levels. For gene expression, ALL, AML, CML and MDS are similar and constitute a cluster. This similarity also results for DLBCL, FL and PTCL, and also for MCL and MZLs. Contrarily, the remaining four HMs (i.e., BL, CLL, HL and MM) hint that specific well-distinct overall gene expression levels characterize them. The scenario slightly changes for the analysis of TFs expression levels. We still observe that ALL, AML, CML and MDS compose a cluster but with the addition of MM, which indicates that these five HMs have a similar overall TFs expression profile. In addition, DLBCL and PTCL still emerge in the same cluster, but they do not turn out to be similar to FL. In contrast, the cluster of MCL and MZLs remains on TFs expression levels, highlighting their similarity. As for specific overall TFs expression levels, we observe similar results compared with the genes scenario, with unique clusters for BL, CLL and HL, while we obtained diverse results in FL. Comparing the two dendograms in Figure 1, we can see how several lymphomas cluster together, such as PTCL with DLBCL, and MZLs with MCL. At the same time, the different types of leukemias are found mutually similar (ALL, AML, CML) and also similar to MDS. These results on lymphomas and leukemias are consistent with the pathophysiology of the HMs.

### 2.2. Hematological Malignancies Indicate Different Over-Expressed Genes and TFs

**Figure 2:**
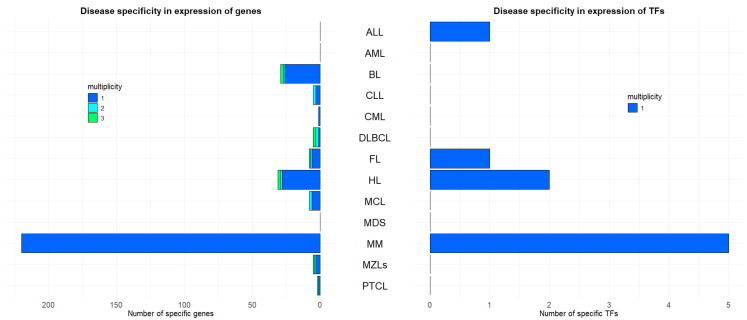
Number and multiplicity of specific genes (**left**) and TFs (**right**) for each HM. The multiplicity of a gene stands for the number of HMs carrying an outlier expression level for that gene.

For each HM, we assess the number of genes and TFs associated with an expression level significantly higher than the median level across all subjects (i.e., p-adj < 0.0.5, and median gene expression greater than the overall subjects median of at least two times the value of the overall inter-quartile range). Figure 2 shows the number of such genes and TFs for each HM, along with their multiplicity, which indicates the number of HMs in which such gene or TFs is significantly over-expressed. We observe that MM carries the largest number of both specific genes (n = 220) and TFs (*BHLHE41*, *ESRRG*, *FOXP2*, *HEY2*, *KLF15*). All lymphomas also report several specific genes, with HL (n = 28) and BL (n = 26) leading, and FL (n = 6), MCL (n = 6), MZLs (n = 3) and PTCL (n = 1) following. Of these, only FL and HL also report a few TF specificities, respectively, one (*SOX9*) and two (*BATF3* and *ISX*). Conversely, leukemias show little specificities with AML showing none overall, ALL reporting only one specific TF (*ZNF423*), and CML a single specific gene (*SFRP1*). Only CLL subjects highlight three specific genes (*ABCA6*, *CLNK*, *PHEX*), whereas, similarly to AML, MDS reports no specificities. The full list of genes is reported in Supplementary Table S4.

**Figure 3:**
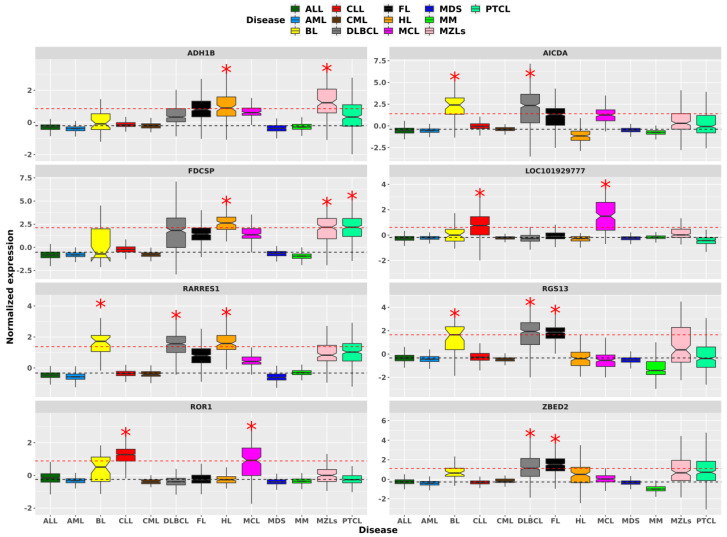
The eight panels display the normalized expression levels of the genes *ADH1B*, *AICDA*, *FDCSP*, *LOC101929777*, *RARRES1*, *RGS13*, *ROR1*, and *ZBED2* (from left to right and top to bottom). These genes show expression levels that significantly deviate from the median per multiple HMs. The black dotted lines represent the population’s median expression level, while the red dotted lines are set at two times the IQR from the median. Red asterisks highlight the HMs where gene expression significantly diverges from the median.

Our findings show that eight genes have a multiplicity greater than one, meaning that eight genes over-expressed in at least two HMs. Figure 3 shows the distribution of the normalized expression levels of these genes: *ADH1B*, *AICDA*, *FDCSP*, *LOC101929777*, *RARRES1*, *RGS13*, *ROR1* and *ZBED2*. Lymphomas are the most represented HMs with BL and DLBCL being the most recurrent ones, emerging exclusively for *AICDA*, HL for RARRES1 and FL for RGS13. Plus, the DLBCL-FL group is also found for *ZBED2*. Another pair of lymphomas, HL and MZLs, is characterized by the significant over-expression of two genes (*ADH1B* and *FDCSP*, which occurs for PTCL as well). Except for groups of lymphomas, only the group of CLL and MCL is pointed out by two genes with multiplicity equal to two (*LOC101929777* and *ROR1*). Ultimately, we observe that over-expressed genes tend mainly to be specific for an HM (i.e., 295 genes with multiplicity of one). Those genes with multiplicity greater than one frequently indicate groups of lymphomas.

### 2.3. Highlighting HMs Similarities and Specificities over Biological Pathways

For each HM we identify significant biological pathways based on their expression levels through Gene Set Enrichment Analysis (GSEA). We discover 57 significantly enriched KEGG pathways, 33 emerging for multiple HMs. We focus in Table 1 on both the single HMs and the groups of HMs that carry a biological relevance in onco-hematology. For the complete list see Supplementary Table S5. As for single HMs, MM shows the highest number of specificities (n = 5), followed by CML (n = 4), ALL and CLL (n = 2). The specific pathways reflect mostly vital cellular (e.g., lysosome and endocytosis) and immune functions (e.g., signaling for B-cells and T-cells). Driving genes for these pathways, which we refer to as those genes whose expression mostly contributes to the significant enrichment, return moderately numerous, i.e., at most 74 genes for the MM regulation of actin cytoskeleton and at least 14 genes for the pentose phosphate pathway of CML. When looking at similarities between HMs, we notice that differently from previous analyses, we can distinguish leukemias. These tend to result in groups of at least three HMs, especially ALL and AML. Among these groups, pathways related to immune response and inflammatory pathways (i.e., chemokine signaling pathway, antigen processing and presentation, complement and coagulation cascades, cytokine receptor interaction and graft versus host disease) indicate a small number of driving genes (n < 12). The five other groups composed by two HMs always include MM and, except for the oxidative phosphorylation pathway, they all point to immune functionalities. We also observe the absence of enriched KEGG pathways with no common driving genes across multiple HMs. That is, not only altered biological functions are similarly identified by a group of HMs, but also their sets of driving genes leading to those pathway alteration.

### 2.4. Hierarchical Clustering on TF-Gene Regulations

**Figure 4:**
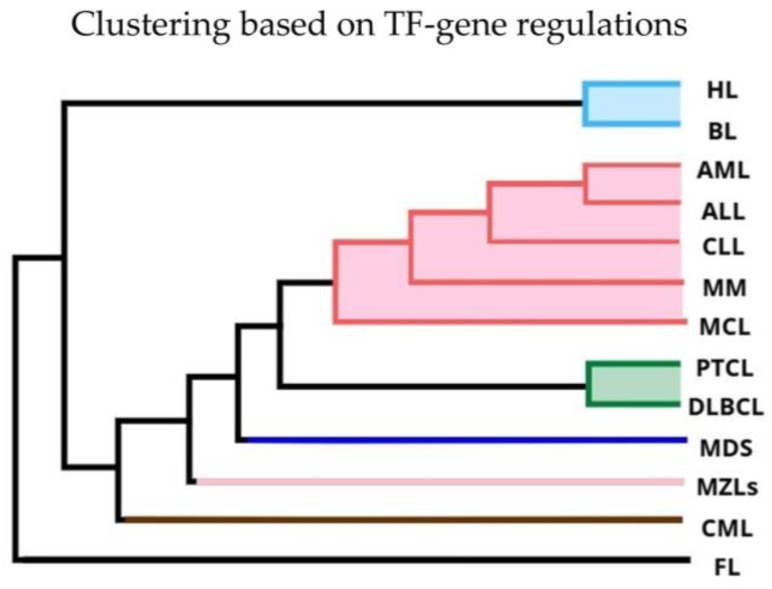
Hierarchical clustering of diseases by similarity score based on TF-gene regulations. The optimal clustering of HMs is represented by the color of the corresponding branches, with black branches indicating single-disease clusters.

We employ GRNs analysis to better understand the relationship between TF regulation, overall gene expression and enriched pathways. To do so, we use PANDA [11,12], an integrative gene regulatory network inference method that models interactions between TFs and their putative target genes to quantify the overall consistency between a TF’s regulatory profile with the target gene’s co-expression. After estimating with PANDA one GRN for each HM, we perform hierarchical clustering on the TF-gene regulations, where each one represents the likelihood that a TF is involved in the regulation of a gene. Figure 4 shows the clustering result over the thirteen HMs. Four HMs (CML, FL, MDS and MZLs) do not join any cluster and, in particular, FL shows to be extremely diverse from all HMs. The remaining nine HMs organize in three clusters: two of them with pairs of lymphomas (BL-HL and DLBCL-PTCL), and one including three leukemias (ALL, AML and CLL) with MM and MCL. Compared with Figure 1, we observe that the cluster of DLBCL and PTCL result on TF-gene regulations as well as they do on gene expression levels (with FL) and TF expression level. The tight connection of AML and ALL is also preserved compared with analyses on expression levels, although the other HMs belonging to their same cluster differ.

### 2.5. TF-Gene Regulations Highlight Fundamental Biological Functions Across HMs

To investigate how the relationship between TFs and genes can affect biological pathways, we perform GSEA on TF-gene regulations. We observe 40 combinations of HMs where the same TF (or TFs) emphasizes the same set of KEGG pathways. Among a total of 100 significant pathways, 54 are enriched in all 13 HMs with at least one same TF. Yet, we also find 16 pathways that result in being affected by one or more TFs only for one specific HM. The comprehensive list of enriched pathways along with the affecting TF can be found in Table S2. In Table 2 we report the exclusively two scenarios where different combinations of HMs enrich pathways that are relevant in onco-hematology and that are indicated by a TF which can be targeted by available drugs [13]. Plus, all the reported pathways associated with HMs pathophysiology through the same TFs also share common driving genes for GSEA, which are listed in the last column of Table 2. Seven pathways are reported in the table, six of which are associated with all diseases. We observe that all pathways are highly interconnected and two TFs appear twice, the CCAAT/Enhancer Binding Protein Beta *CEBPB* and the Nuclear Factor Erythroid 2 Like 1 *NFE2L1*, suggesting that the significant process alteration observed in the disease not only can be traced back to shred pathways but also to overlapping sets of genes and TFs.

## 3. Discussion

### 3.1. Hierarchical Clustering Based on Gene Expression and TF Shows Divergent Clustering in Leukemias and Lymphomas

The hierarchical clustering analysis based on gene and TF expression levels provides insightful distinctions among HMs. The clustering reveals the presence of a leukemic cluster, involving ALL, AML, CML, and MDS, that consistently group together, indicating similar gene expression profiles. This clustering is consistent across both gene expression and TF expression levels, with the addition of MM in the leukemic cluster in the TF expression analysis. The clustering of DLBCL and PTCL, and the consistent grouping of MCL and MZLs, identifies a second cluster made predominantly of Lymphomas, highlighting similarities in their expression profiles. However, the distinct clustering of BL, CLL, HL, and MM based on overall gene expression levels suggests unique signatures that differentiate these HMs from others. The comparison of dendrograms for gene expression and TF expression levels highlights subtle differences in the transcriptomic regulation of HMs. The inclusion of MM in the cluster with ALL, AML, CML, and MDS in the TF expression analysis shows that TFs may play a crucial role in the pathophysiology of MM with shared underlying mechanisms with leukemias, as other functional and epidemiological studies already suggest [13,14]. The contrast between observations based on gene expression levels—where MM forms a distinct cluster but is more closely associated with lymphomas—and TF-based clustering may also be due to the complex structural genetics of MM [15,16], including factors like translocations, recombinations, and hyperdiploidy, which can influence similarities when comparing overall gene expression versus TFs expression alone. Overall, these findings are consistent with the known pathophysiology of HMs, where leukemias (ALL, AML, CML) and MDS share common molecular features, and certain lymphomas (PTCL, DLBCL, MCL, MZLs) exhibit similar expression profiles [17]. The separation of FL from DLBCL and PTCL in the TF expression analysis, despite their clustering in the gene expression analysis, indicates that TF expression provides additional layers of differentiation not captured by gene expression alone [18,19]. The distinct expression patterns observed in BL, CLL, HL, and MM highlight the heterogeneity within HMs and underscore the importance of considering both gene and TF expression levels in understanding their molecular underpinnings. This comprehensive analysis enhances our understanding of the molecular landscape of HMs and may inform targeted therapeutic strategies.

### 3.2. Gene and Transcription Factor Expression Highlight Unique and Overlapping Traits Among HMs

In our search for evidence to explain HM clusters based on disease-specific gene expression and TFs, we observe a lack of specificity. Only a minimal fraction (at most 0.9%) of genes and TFs expression levels are disease-specific traits. This could be due to the shared tissue-specific nature of these blood-related diseases. This is also the most plausible reason for the small level of multiplicity (at most three) we find for disease-specific genes [20]. Disease-specific genes are especially informative for single clusters. MM showed the highest number of specific genes and TFs, indicating a distinct expression profile. Among 220 MM-specific genes, *IGF1* [16], *SLAMF7* [21], *ITGA8* [22], *IL5RA* [23], *TJPI* [24], *GPRC5D* [25], *DKK1* [26], and *WNT5A* [27] harbor known significance for MM, whereas most others have weaker connections to the disease. However, this abundance of MM-specific genes can explain its unique clustering. Among lymphomas, HL and BL showed the most specific genes, with HL having unique TFs like *BATF3* and *ISX1*. In contrast, leukemias displayed fewer specificities, with AML showing none, and CLL highlighting five specific genes, two of which overlap with MCL. Moreover, single clusters for HL and BL correlate with their respective number of disease-specific genes. However, explaining the clustering of CLL is more challenging, as only three CLL-specific genes (*PHEX*, *CLNK*, *ABCA6*) were identified, none of which are directly associated with the disease. This contrasts with HL- (*CCL17*, *CCL22* [28], *IL13* [29], *CCL13* [30]) and BL- (*ZNF385B* [31] and *UCHL1* [32]) specific genes, which carry known disease-related information. Additionally, CML, DLBCL, FL, MCL and PTCL also have specific genes with multiplicity one and some of them are also recognized to have impact on prognosis and treatment (e.g., *SFRP1* for CML [33], *NUPR1* for DLBCL [34], *HTR3A* and *IGH* for FL [35,36], *SOX11* for MCL [37]). Regarding disease-specific TFs, the specificity analysis shows interesting results, with MM again standing out as the HM with the most specific TFs. All diseases with specificities, except FL, highlight known TFs for their diseases, i.e., *ZNF423* for ALL [38], *BATF3* for HL [39] and *BHLHE41* for MM [40]. Therefore, the clustering outcome from the specificity analysis competes with the hierarchical tree clusters rather than validating them. Such signatures identify candidate genes sharing expression levels out of the ordinary level for all HMs. Among the seven HMs groups, BL and DLBCL share the lymphomagenesis-related gene *AICDA* [41], and CLL and MCL are grouped by *ROR1*, which is known to impact disease progression in CLL patients [42]. This suggests that some of the other five HMs groups may have clinical or pathobiological relevance. Our analysis shows that TF expression tends to be specific to a single HM, whereas overall gene expression shows more similarities among multiple HMs [43]. These findings suggest potential common underlying TF expression alterations between these diseases. This detailed mapping of gene and TF specificities improves the current understanding of the transcriptional regulation of HMs and the importance of considering both unique and shared expression traits in disease characterization and treatment development.

### 3.3. Different HMs Commonly Emphasize the Same Biological Pathways

The combination of expected and novel signatures across HMs led us to explore a higher biological context linked with expression. We believe HMs cluster based on many similar gene expression levels rather than a few highly divergent ones. GSEA results support this, identifying 57 significantly enriched KEGG pathways, with 33 pathways emerging across multiple HMs. Notably, we observe the following: (i) the unique clustering of MM on eight enriched pathways, and (ii) the distance between CLL and ALL, possibly explaining why CLL doesn’t cluster with leukemias. Our work identified three HM groups sharing enrichment in the same biological pathways, suggesting shared molecular mechanisms. Despite distinct clinical presentations, leukemias may share underlying biological processes with other HMs. All emerging signatures from gene expression levels and KEGG pathways help unravel early hierarchical clusters, providing complementary insights. GSEA analyses shift our focus to biological function rather than gene signatures. We then focused on the genes that significantly determine pathway enrichment, providing a deeper insight into the molecular basis of pathway alterations. Our ultimate interest in drug repurposing leads us to explore those genes in the enriched pathways. We focused on genes that both (i) drive the gene set enrichment task for all HMs within the cluster, and (ii) are already druggable, finding seven cellular signaling pathways respectively through *CEBPB*, *EPAS1*, *NFE2L1*, *NFKB2*, *NR4A3*. Two TFs appear twice, the CCAAT/Enhancer Binding Protein Beta *CEBPB* and the Nuclear Factor Erythroid 2 Like 1 *NFE2L1. CEBPB* works in tandem with another transcription factor, *MYB* and the co-activator *p300*, to regulate the expression of genes essential for the proliferation and survival of AML cells [44,45]. *CEBPB* was described to have a pro-oncogenic role also in other HMs, such as anaplastic large cell lymphoma (ALCL)[46] as well as in different cancers, e.g., breast, colon, kidney, stomach, prostate, and ovaries [47,48]. *CEBPB* is currently being considered as an interesting drug target in AML, and molecules like sesquiterpene lactones (STLs) [44] and helenalin acetate (HA) [47] have shown promising results in disrupting the pro-oncogenic activity of the *CEBPB/MYB/p300* complex by targeting directly *CEBPB. NFE2L1* codes for the TF NRF1, which plays crucial roles in proteostasis, and particularly in the so called “proteasome bounce-back response”. This recovery response has medical significance as some therapeutic proteasome inhibition therapies trigger the upregulation of proteasome genes in cancer cells, such as in MM, leading to anticancer drugs resistance [49,50]. Our combination of regulating TFs, biological context, and driving genes highlights known factors of HMs. Our results summarize TF-gene regulations and biological functions into possible signatures. Targeting shared pathways and TFs could be a viable strategy for broad-spectrum therapies for HMs. The overlap of transcriptomic alterations suggests that targeted therapies could address multiple HMs by focusing on common pathways and driving genes, leading to more effective treatments. Our results identify many driving drug targets, and we propose that analyzing regulatory networks can help pinpoint putative drug targets more precisely.

### 3.4. Distinct Regulatory Patterns Emerge Across HMs

HMs with similar patterns of gene expression levels may not be orchestrated by similar gene regulation patterns throughout the entire regulatory network [51]. Gene expression levels are often used as a proxy for regulation, but they only reflect the net effect of regulatory interactions. To understand the interplay of these aspects in HMs, we analyzed regulatory networks with PANDA. We observed hierarchical clusters and HM-specific TF-gene regulations. Four HMs (CML, FL, MDS, and MZLs) did not cluster with other diseases, while the remaining nine HMs formed three clusters: two pairs of lymphomas (BL-HL and DLBCL-PTCL) and a cluster of three leukemias (ALL, AML, and CLL) with MM and MCL. This clustering aligns with previous gene expression analyses. About 20% of TF-gene regulations are HM-specific, suggesting TF-gene regulations better distinguish diseases than HM-specific genes. FL stands out in TF-gene regulations, similar to how MM stands out in gene expression. The numerous HM-specific TF-gene regulations require further study to identify fine discriminant TF-gene regulations across HMs. Previous research has significantly advanced our understanding of cancer classification and gene regulatory networks in hematopoietic cancers. The seminal work of Golub et al. [4] introduced a groundbreaking approach using microarrays to classify cancers based on gene expression profiles. This approach laid the foundation for more precise and personalized cancer treatments by identifying specific molecular markers for different tumor types. More recently, network approaches have been applied in the field of HM through gene co-expression networks (GCNs), describing the prevalence of intra-chromosomal interactions and the presence of overexpressed pseudogenes in AML [52]. This work highlighted the distinct regulatory landscapes of hematopoietic cancers and emphasized the importance of understanding these networks for better disease detection and treatment. GNR analysis has been successfully applied to decipher tissue-specific gene regulation and more in general pathological conditions and aging [11,53,54]. Building on these foundational studies, our work on TF-gene regulation expands the current state of the art by integrating gene regulatory networks to model interactions between TFs and their target genes across HMs.

## 4. Conclusions

Our study provides a detailed exploration of GRNs in HMs using publicly available microarray data. We identified distinct patterns of gene and TF expression among various HMs. The clustering analysis revealed a clear separation between leukemias and lymphomas, with specific clusters highlighting similarities and differences in their molecular profiles. Notably, MM exhibited unique clustering patterns. The gene and TF expression analysis underscored the importance of considering both unique and overlapping traits in understanding HMs. The identification of disease-specific genes and TFs provides valuable insights into the molecular features of these malignancies and highlights potential targets for therapeutic intervention. GSEA further supported our findings by identifying 57 significantly enriched KEGG pathways, with 33 pathways shared across multiple HMs. The identification of druggable genes within these pathways, particularly those regulated by TFs such as CEBPB and NFE2L1, offers promising targets for drug repurposing and the development of broad-spectrum therapies. Our network analysis revealed that TF-gene regulations tend to better distinguish HMs compared to gene expression alone. The significant overlap of transcriptomic alterations across HMs suggests that targeting shared pathways and TFs could lead to more effective and comprehensive treatment strategies. However, our findings suggest rather than prove and need further validation. Here, we avoid using in silico validation to have enough representativeness for all HMs. This is especially relevant for rare HMs that are not numerous but still heterogeneous (i.e., BL, HL and CML). Although beyond of the scope of this work, we believe that in vitro validation of our findings using cell culture is an important direction for future research. Ultimately, our study contributes to exploring the transcriptional landscape of HMs and provides candidate targets to perform future pre-clinical validation, which can further help to identify novel therapeutic targets based on either differentially expressed genes or TF-gene interactions.

## 5. Materials and Methods

### 5.1. Dataset Pre-Processing and Batch Correction

We collected microarray data from 34 datasets available on the Gene Expression Omnibus (GEO), listed in Supplementary Table S3. All microarrays were acquired by the Affymetrix Human Genome U133 Plus 2.0 platform [55]. Healthy subjects do not participate in the analysis alongside pediatric patients (younger than 18). Exclusively untreated subjects take part in the analyses and no subject has replicas in the dataset. We combined three types of lymphoma, i.e., mucosa-associated lymphoid tissue (MALT), splenic marginal zone lymphoma (SMZL), and marginal zone lymphoma (MZL), to form a single type of lymphoma (MZLs). Quality control and filtering are carried out first via image assessment and then via thresholding on GNUSE values [56]. The fRMA approach [57] is used to perform background correction, normalization, and summarization, resulting in a final cohort of 5442 patients. A flowchart representing the complete filtering procedure is given in the Supplementary Figure S3. Principal Component Analysis (PCA) showed the presence of systematic variability due to the data providers. We account for batch effect by first estimating negative controls (i.e., genes with no expected biological interplay) and then employing RUV [58]. Further details can be found in the Supplementary Materials. We run our procedure on the entire dataset, as we intend to preserve the heterogeneity across HMs. No in silico validation (e.g., cross-validation) is therefore performed at this stage or in the following ones.

### 5.2. Analyses at Gene Expression Levels

Expression data are available for 19901 genes and 609 TFs. The complete list of these TFs is available in Table S3. Firstly, we want to estimate a common measure for the expression level for each gene per HM. To this end, we compute the following score [7] for each gene:

$$s_{j}^{dis}\;=\;\frac{med\left(e_{j}^{dis}\right)\;-\;med\left(e_{j}\right)}{IQR\left(e_{j}\right)}$$

where $e_{j}$ indicates the expression level for the *j*-th gene. This score quantifies how strongly the median expression of a gene or TF in the subset of subjects specific to each disease differs from its median value over all the HMs, accounting for its intrinsic variability (i.e., IQR). Given that most genes are not expected to be disease-specific, the distribution of all scores follows a Gaussian distribution roughly centered around zero (μ≈0.002 and σ≈0.363). This is similar for TFs (μ≈0.009 and σ≈0.375). From this empirical distribution we compute a *p*-value for each score and we adjust them for multiple testing. We define specific genes or TFs as those with their score larger than 2 (i.e., $s_{j}^{dis}\;>2$) and *p*-value smaller than 0.01. That is, we consider as specific only genes and TFs that greatly significantly over-express within an HM compared to all the thirteen HMs. As a final step, we count the multiplicity of each gene. This is the number of HMs showing specificity for that gene.

We define a distance metric over the scores of expression levels that is needed to perform hierarchical clustering. Given diseases A and B, we compute all pairwise Euclidean distances across the scores. Next, we derive a dendrogram using the complete linkage method [59]. We obtain the ultimate hierarchical clustering of the HMs by cutting the dendrogram via adaptive branch pruning [60].

Unlike the other two analyses, we perform GSEA for each HM using the median expression level. We adopt the FGSEA [61] algorithm for all GSEAs and we select pathways from the KEGG database [62]. To account for multiple testing we gather all results of GSEA across HMs and we correct *p*-values via Benjamini-Hochberg [63]. The number of tests performed are the number of pathways of KEGG (186) times the number of HMs (13), i.e., 2418. We consider as significative a pathway with a *p*-value < 0.01.

### 5.3. Analyses on TF-Gene Regulations

From expression profiles, we obtain a gene-to-gene Pearson correlation matrix for each HM. Interpreting the gene-to-gene correlation matrix as adjacency matrix of a weighted, symmetric network, we generate thirteen gene-to-gene disease-specific co-expression networks. Next, we employ PANDA [11] to estimate generate regulatory networks. PANDA is an integrative gene regulatory network inference method, which models the intricate regulatory processes by considering interactions between TFs and their target genes. Further details on PANDA can be found in Section S12 of Supplementary Materials. This yields thirteen bipartite graphs modelling regulatory networks for all HMs. At this point, we reproduce analyses in line with what we perform on expression levels. We define disease-specific regulations, i.e., links between a TF and a gene, by using a score formulated as follows:

$$s_{ij}^{dis}\;=\;\frac{e_{ij}^{dis}\;-\;med\left(e_{ij}\right)}{IQR\left(e_{ij}\right)}$$

where the median value of a regulation across all 13 disease-specific regulatory networks is subtracted from the value of a regulation for a certain HM and is then divided by the overall IQR. Like for expression levels, this score measures the deviance of a regulation with respect to all HMs. Even for regulations, most of them are not expected to be disease-specific and the scores distribute as a Gaussian centered around zero (μ≈0.002 and σ≈0.973). We calculate a *p*-value for all scores, and we consider regulations to be disease-specific when they have *p*-value lower than 0.01 and score larger than 2. It follows that we determine the pairwise distance between HMs as the Euclidean distance including all regulations. Thereafter, we run the hierarchical clustering over the distances based on the complete linkage method and we adaptively pruned the dendrogram to get the optimal clusters. Since each regulatory network is a weighted graph, each TF carry one weight for every gene. We then perform GSEA within each disease for all TFs independently. This yields a total of 1.5 M tests; that is, 609 times (i.e., the number of TFs) more numerous than the GSEA tests we face in the analyses on expression levels. Owing to this high increment of tests, after adjusting all *p*-values by Benjamini-Hochberg, we place the threshold of significance in the results at 0.05.

### 5.4. Intersection-Based Signatures for Clustering and Targets Prioritization

We observe that multiple HMs report the same over-expressed genes. Thus, we cluster these diseases and define the set of their shared over-expressed genes as their signature. We did the same for TF-gene regulations and pathways enriched by expression levels. As for the enriched pathways, we examine which genes lead to the significant enrichment and we call driving genes those that mostly contribute. Noteworthy, in pathways analysis over TF-gene regulations, multiple HMs can enrich the same pathway through the same or different TFs, with driving genes potentially changing. To dilute these outcomes, we only form clusters of HMs that share the (i) same enriched pathways, (ii) TFs, and (iii) at least one common driving gene. As seen in the past [64], we find this intersection-based approach improves the interpretability of the results, since it strictly narrows them down. In this work, when many genes result for a certain analysis, we prioritize the report of those genes that either (a) carry a known value for a pathology, prioritizing neoplasies, or (b) are associated with existing drugs. To take advantage of such knowledge we employ DisGeNet [65] and DrugBank [66].

## Figures and Tables

**Table 1:** Organization of the HMs based on commonly enriched biological functions. Results from GSEA for each disease show that several KEGG pathways are enriched across multiple diseases. Next to each pathway, we report between brackets the number of driving genes, which indicate the genes mainly involved in the enrichment of a pathway. Green-shaded cells in the table indicate specificities, while the blue-shaded cell represents similarities.

Hematological Malignancies	Enriched KEGG Pathways
ALL	Lysosome (41), DNA replication (21)
CLL	Purine metabolism (49), B-cell receptor signaling pathway (37)
CML	Endocytosis (58), Insulin signaling pathway (44), Erbb signaling pathway (24), Pentose phosphate pathway (14)
MM	Regulation of actin cytoskeleton (74), Fc gamma r mediated phagocytosis (47), T-cell receptor signaling pathway (45), Fc epsilon RI signaling pathway (30), Nod like receptor signaling pathway (28)
CLL, MM	Natural killer cell mediated cytotoxicity (22)
CML, MM	Primary immunodeficiency (7)
DLBCL, MM	Leukocyte transendothelial migration (12)
MDS, MM	Hematopoietic cell lineage (17)
MM, MZLs	Oxidative phosphorylation (35)
ALL, MZLs, PTCL	Ribosome (39)
ALL, AML, FL, MDS, MZLs	Cell cycle (21)
ALL, AML, MDS, MM, MZLs	Spliceosome (30)
ALL, AML, BL, DLBCL, MCL, MDS, MZLs, PTCL	Focal adhesion (22)
ALL, DLBCL, FL, HL, MCL, MM, MZLs, PTCL	Chemokine signaling pathway (8)
AML, CML, DLBCL, FL, HL, MDS, MM, PTCL	Antigen processing and presentation (5)
AML, CLL, CML, FL, HL, MDS, MM, MZLs, PTCL	Graft versus host disease (5)
ALL, AML, BL, CML, DLBCL, HL, MCL, MDS, MZLs, PTCL	ECM receptor interaction (12)
ALL, AML, BL, DLBCL, FL, HL, MCL, MDS, MZLs, PTCL	Complement and coagulation cascades (12)
ALL, AML, DLBCL, FL, HL, MCL, MDS, MM, MZLs, PTCL	Cytokine cytokine receptor interaction (9)
AML, CLL, CML, DLBCL, FL, HL, MCL, MDS, MM, MZLs, PTCL	Cell adhesion molecules cams (5), Allograft rejection (4)

**Table 2:** Combinations of multiple HMs based on identical enriched pathways obtained from the same TF-gene regulation on all genes. KEGG pathways are reported with their complete name along with their corresponding KEGG ID. The column of driving genes relates to those genes that in all diseases in the cluster lead the GSEA to the enrichment of the pathways.

HMs	Enriched Pathway	TF	Common Driving Genes
ALL, AML, BL, CLL, CML, DLBCL, FL, HL, MCL, MDS, MM, MZLs, PTCL	AMINOACYL TRNA BIOSYNTHESIS (hsa00970)	CEBPB	*AARS*, *CARS*, *EPRS*, *FARSA*, *GARS*, *HARS* and other 10 genes
	MAPK SIGNALING PATHWAY (hsa04010)	EPAS1	*AKT1*, *AKT2*, *BDNF*, *CACNB4*, *FGFR1*, *FGFR4*, *MAP2K2*, *MAPT*, *NTRK2*, *TGFB1* and other 50 genes
	CELL CYCLE (hsa04110)	NFE2L1	*CDKN1A*, *CHEK1*, *CHEK2*, *MDM2*, *MYC*, *SMAD2*, *TP53*, *WEE1* and other 22 genes
	UBIQUITIN MEDIATED PROTEOLYSIS (hsa04120)	NFE2L1	*MDM2*, *XIAP* and other 33 genes
	CHEMOKINE SIGNALING PATHWAY (hsa04062)	NFKB2	*AKT2*, *PIK3CD*, *PIK3R1*, *PRKACA*, *PRKCD* and other 12 genes
	FOCAL ADHESION (hsa04510)	NR4A3	*COL2A1*, *ERBB2*, *FLNA*, *FLT4*, *IGF1R*, *LAMA1*, *PDGFRA*, *PDGFRB*, *PIK3R1*, *VEGFA* and other 50 genes
AML, CLL, HL, MCL, PTCL	ABC TRANSPORTERS (hsa02010)	CEBPB	*ABCC6*, *CFTR*, *ABCC1*, *ABCD3*, *ABCC8*, *ABCC3*,*ABCG1*, *ABCC5*, *ABCC4*, *ABCA8*, *ABCB9*, *ABCA6*, *ABCA5*, *ABCC10*, *ABCA13*

## Data Availability

The 36 datasets supporting the conclusions of this article are available in the GEO Database. IDs of each dataset used in this work are reported in Supplementary Materials. Both original and batched-corrected datasets are available upon request from the corresponding author.

## Supplementary Materials

The following supporting information can be downloaded at: https://www.mdpi.com/article/10.3390/ijms252413588/s1. References [7,67,68,69,70] are cited in the supplementary materials.
