# Supplementary material for: Emerging Signatures of Hematological Malignancies from Gene Expression and Transcription Factor-Gene Regulations
Source: Int J Mol Sci. 2024 Dec 19;25(24):13588. doi: 10.3390/ijms252413588 (PMC11678896; doi:10.3390/ijms252413588)
Supplement: Supplementary file 1 [file ijms-25-13588-s001.zip › ijms-3002846-supplementary.pdf]

International Journal of *Molecular Sciences*

Article

Emerging Signatures of Hematological Malignancies from Gene Expression and Transcription Factor-Gene Regulations

Daniele Dall'Olio [1,†], Federico Magnani [1,†], Francesco Casadei [2], Tommaso Matteuzzi [3], Nico Curti [4], Alessandra Merlotti [4], Giorgia Simonetti [5], Matteo Giovanni Della Porta [6], Daniel Remondini [4], Martina Tarozzi [1,7,‡] and Gastone Castellani [1,7,*,‡]

[1] Department of Medical and Surgical Sciences, University of Bologna, 40138 Bologna, Italy
[2] IRCCS Istituto delle Scienze Neurologiche di Bologna, 40139 Bologna, Italy
[3] Department of Physics and Astronomy, University of Firenze, 50019 Sesto Fiorentino, Italy
[4] Department of Physics and Astronomy, University of Bologna, 40127 Bologna, Italy;
[5] Biosciences Laboratory, IRCCS Istituto Romagnolo per lo Studio dei Tumori (IRST) "Dino Amadori", 47014 Meldola, Italy
[6] Comprehensive Cancer Center, IRCCS Humanitas Clinical and Research Center and Department of Biomedical Sciences, Humanitas University, 20089 Milan, Italy
[7] IRCCS Azienda Ospedaliero-Universitaria di Bologna, 40138 Bologna, Italy
* Correspondence: gastone.castellani@unibo.it
† These authors equally contributed to this work.
‡ These authors equally contributed to this work.

S1. Acronyms for HMs (Hematological Malignancies)

**ALL**	Acute Lymphocytic Leukemia
**AML**	Acute Myeloid Leukemia
**BL**	Burkitt Lymphoma
**CLL**	Chronic Lymphocytic Leukemia
**CML**	Chronic Myeloid Leukemia
**DLBCL**	Diffuse Large B-Cell Lymphoma
**FL**	Follicular Lymphoma
**HL**	Hodgkin Lymphoma
**MCL**	Mantle Cell Lymphoma
**MDS**	Myelo-Dysplastic Syndromes
**MM**	Multiple Myeloma
**MZLs**	Marginal Zone Lymphomas
**PTCL**	Peripheral T-Cell Lymphoma

Supplementary Table S1. *List of the thirteen HMs considered in this study, along with their acronyms.*

S2. Overview of available HMs

HM	Subjects
**ALL**	730
**AML**	1230
**BL**	38

 https://doi.org/10.3390/ijms252413588

**CLL**	862
**CML**	115
**DLBCL**	417
**FL**	452
**HL**	98
**MCL**	158
**MDS**	338
**MM**	595
**MZLs**	138
**PTCL**	271

Supplementary Table S2. *Number of microarray samples available for each HM. Since there are no patient replicates in the dataset, that is also equal to the number of subjects.*

S3. Distribution of sources across HMs

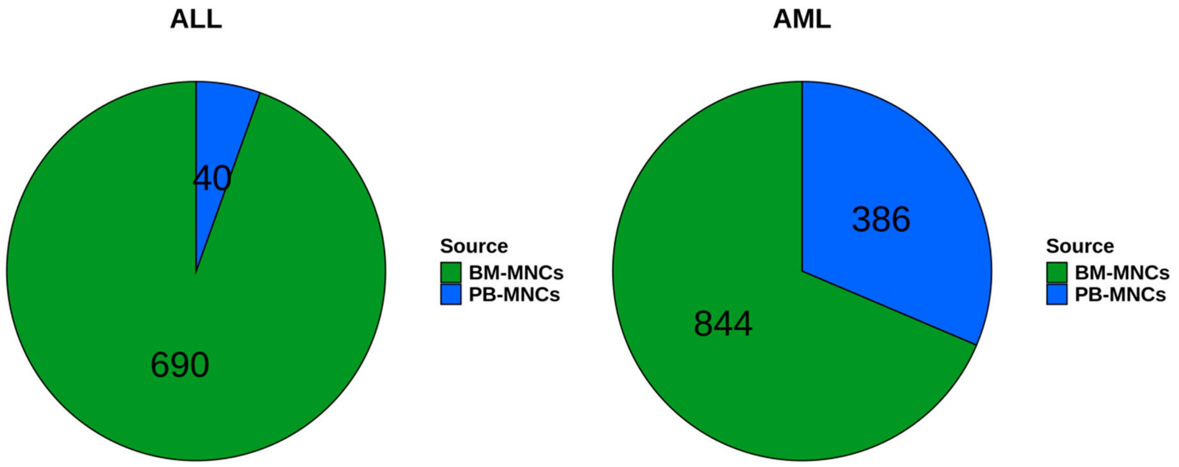

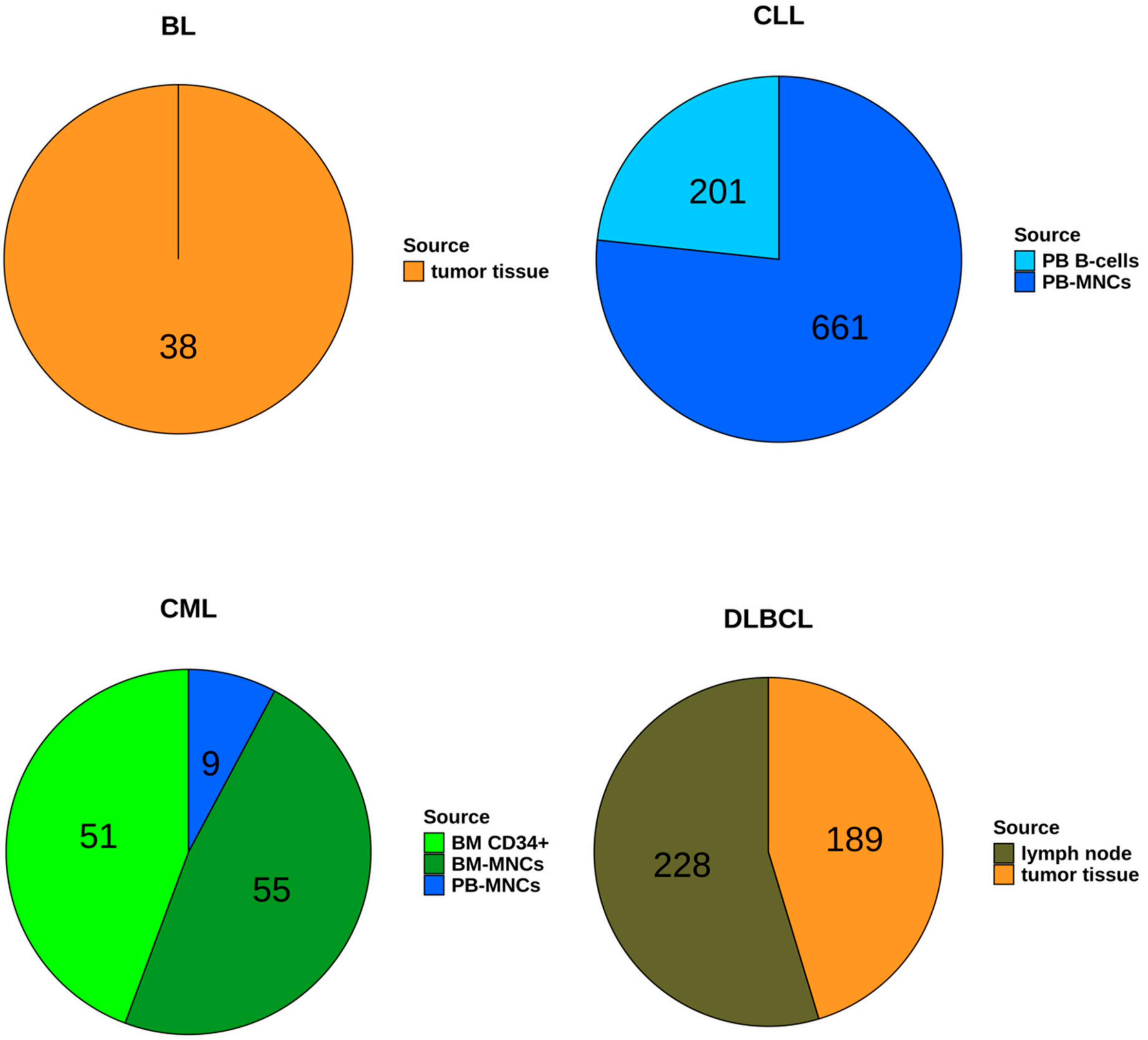
BL
38
Source
tumor tissue
CLL
201
661
Source
PB B-cells
PB-MNCs
CML
9
51
55
Source
BM CD34+
BM-MNCs
PB-MNCs
DLBCL
228
189
Source
lymph node
tumor tissue

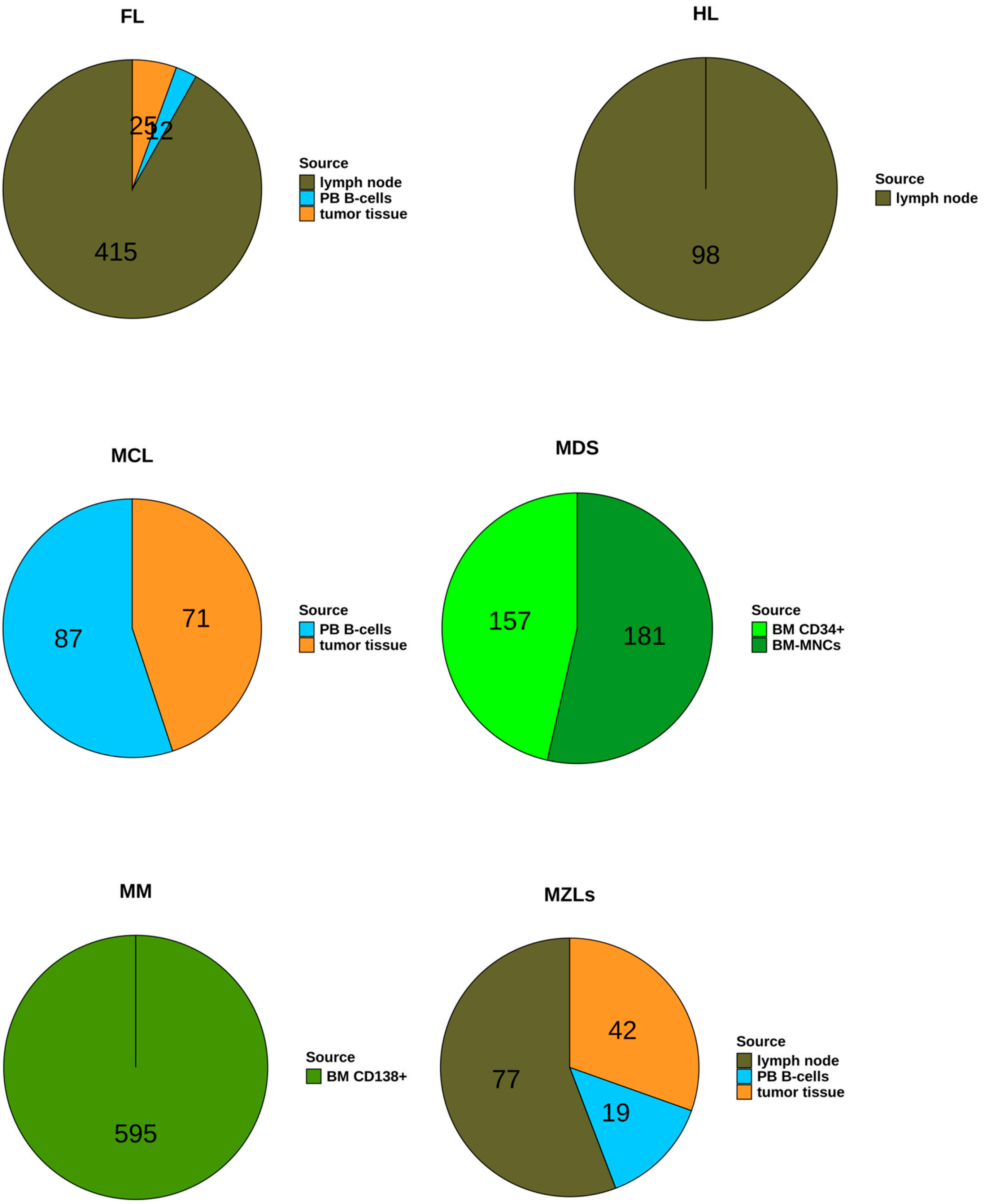
FL
25
12
415
Source
lymph node
PB B-cells
tumor tissue
HL
98
Source
lymph node
MCL
87
71
Source
PB B-cells
tumor tissue
MDS
157
181
Source
BM CD34+
BM-MNCs
MM
595
Source
BM CD138+
MZLs
42
77
19
Source
lymph node
PB B-cells
tumor tissue

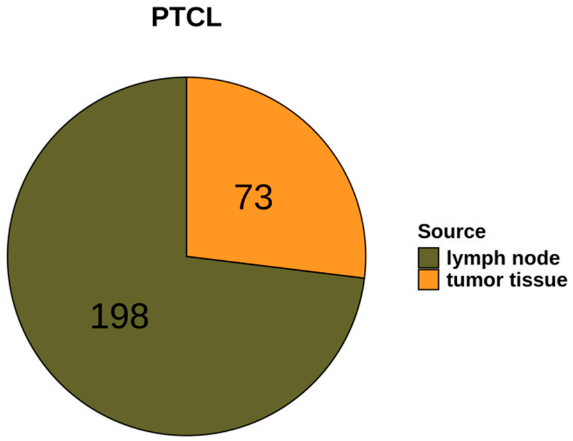

Supplementary Figure S1. *Biological sources where HMs samples are extracted from. Seven sources (lymph node, BM-MNCs, PB-MNCs, BM CD34+, VM CD 138+, PB B-cells, tumor tissue) result with many of them being the tissue required for diagnosis.*

S4. Methods of tissue preservation over HMs

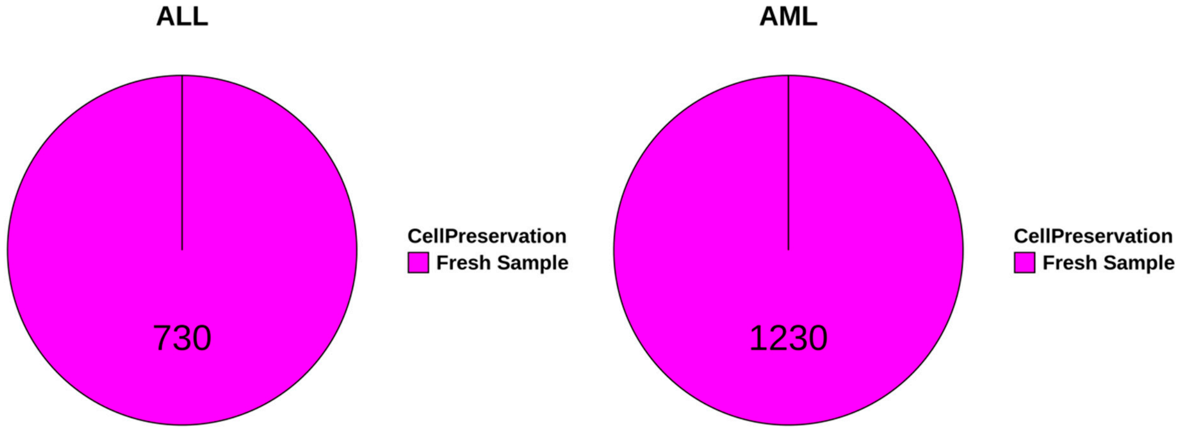

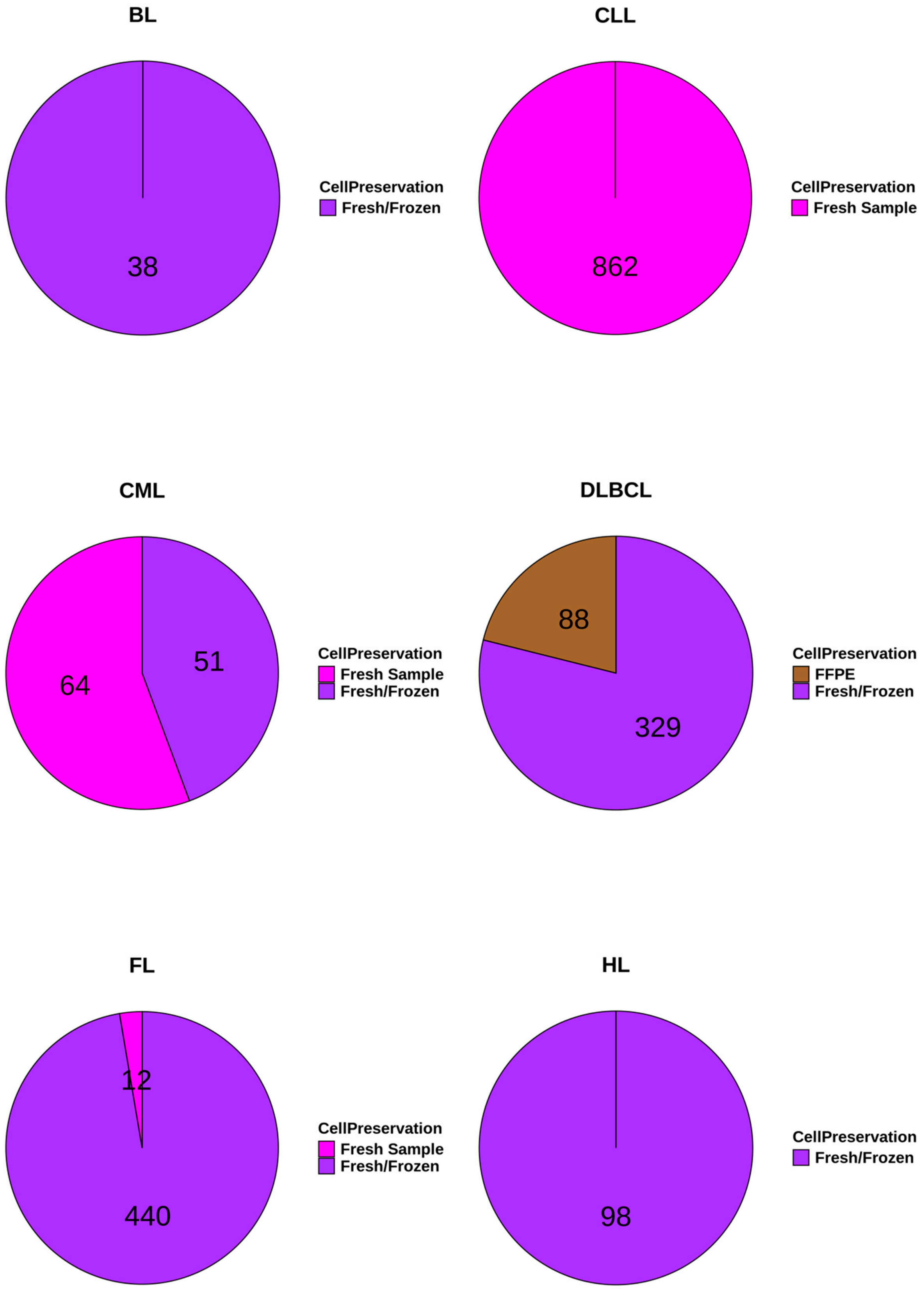
BL
38
CellPreservation
Fresh/Frozen
CLL
862
CellPreservation
Fresh Sample
CML
64
51
CellPreservation
Fresh Sample
Fresh/Frozen
DLBCL
88
329
CellPreservation
FFPE
Fresh/Frozen
FL
12
440
CellPreservation
Fresh Sample
Fresh/Frozen
HL
98
CellPreservation
Fresh/Frozen

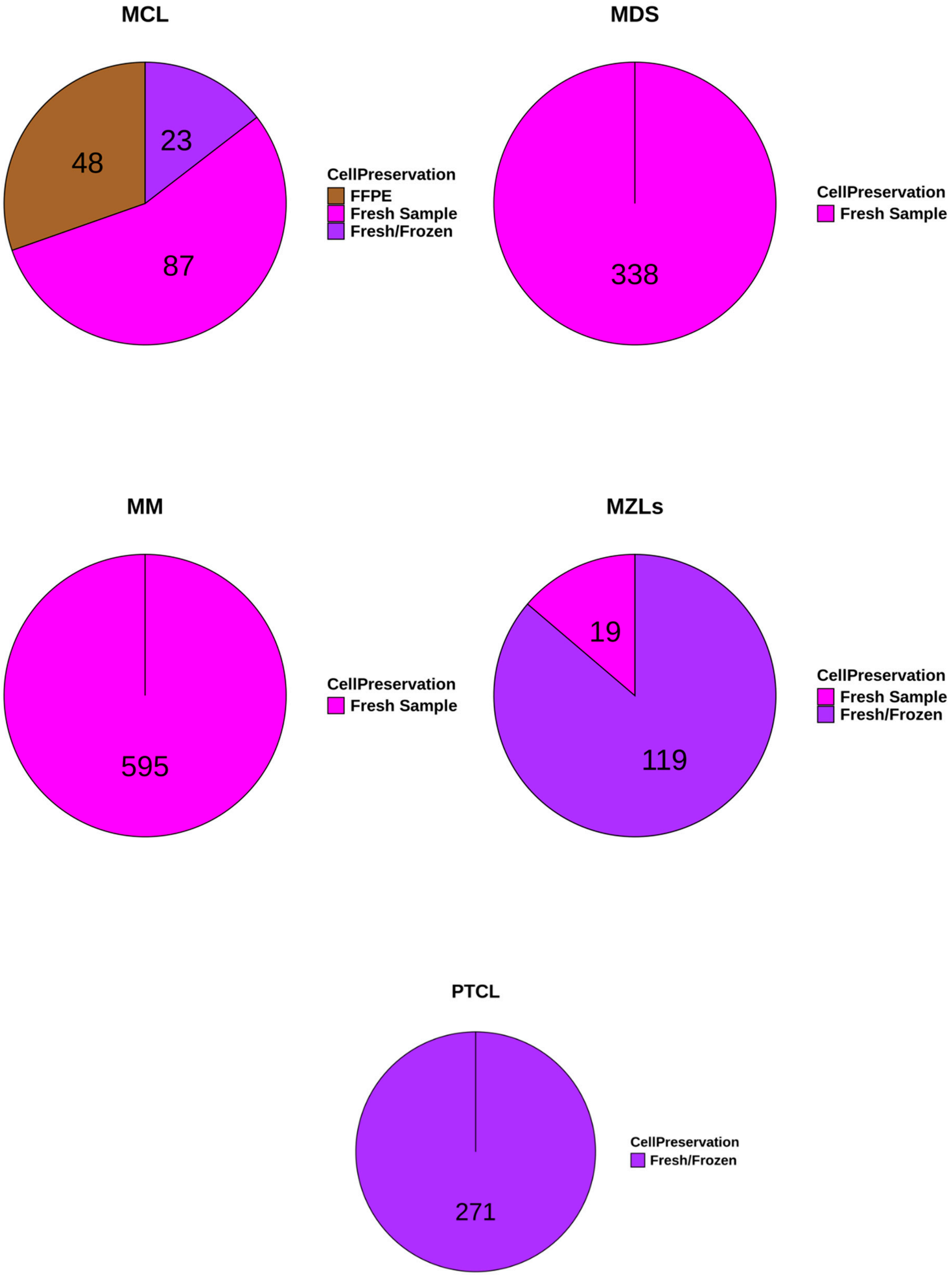

Supplementary Figure S2. *Methods of tissue preservation over all HMs. Three general types are reported: either fresh or frozen tissue, fresh tissue, and FFPE-preserved tissue. Most HMs derive from either fresh or frozen tissue, which better conserve the signal for transcriptomics. A*

small number of FFPE samples are also included in this work. To be noted, the batch-correction steps involved in our analyses should filter out bias owed to the preservation methods.

S5. Data selection and cleaning

In this work we exclusively analyze publicly available data from Gene Expression Omnibus (GEO). We select microarray data to estimate gene expression levels and we thoroughly choose to collect only experiments using the Affymetrix Human Genome U133 Plus 2.0 platform. We exclude two blood-related non-cancerous conditions, myelofibrosis (AMM) and monoclonal gammopathy of undetermined significance (MGUS), upon clinical indications and we consider three types of lymphoma, i.e., mucosa associated lymphoid tissue (MALT), splenic marginal zone lymphoma (SMZL) and marginal zone lymphoma (MZL), as a single kind of HM, which we refer to as MZLs. Our interest in adult subjects suffering from any of the thirteen HMs also requires taking out (i) samples from healthy subjects and (ii) pediatric cases, i.e., under eighteen years old. Plus, we do not consider (iii) possible subject replicas and (iv) we focus only on samples from untreated subjects.

S6. Pre-processing

We perform quality control and filtering sequentially first through a visual inspection of images and then using a cut-off on GNUSE values. We remove subjects whose microarray image is characterized by either scratches, hazes, or unusual spots. This results in the removal of 1563 subjects, who also contain all DLBCL subjects coming from dataset GSE31312. Subsequently, we utilize the fRMA approach to perform background-correction, normalization, and summarization. Following, we threshold GNUSE at 1.25 and we discard 404 subjects. Doing so, another entire dataset (GSE79533) turns out to be neglected. This recommended GNUSE threshold indicates that the variability of a subject intensity values should not exceed the size of the first quartile of the median subject intensity distribution. We eventually are left with 5442 subjects over 34 datasets. The complete collection of these datasets is reported below, together with a flowchart (Supplementary Figure S3) representing the major cleaning actions applied to the dataset.

GEO Dataset
GSE14671
GSE39133
GSE12195
GSE118238
GSE19069
GSE66006
GSE93291
GSE36000
GSE25550
GSE24080
GSE53786
GSE15434

GSE19429
GSE35426
GSE127462
GSE79196
GSE58445
GSE21261
GSE6338
GSE17920
GSE11318
GSE132929
GSE35348
GSE39577
GSE13314
GSE69034
GSE39671
GSE12417
GSE19784
GSE34171
GSE6891
GSE93261
GSE50006
GSE13159

Supplementary Table S3. *List of all 34 GEO datasets providing data for this work. Yet not all data coming from such datasets take part in the analyses. Subjects with low-quality samples are in fact removed during pre-processing.*

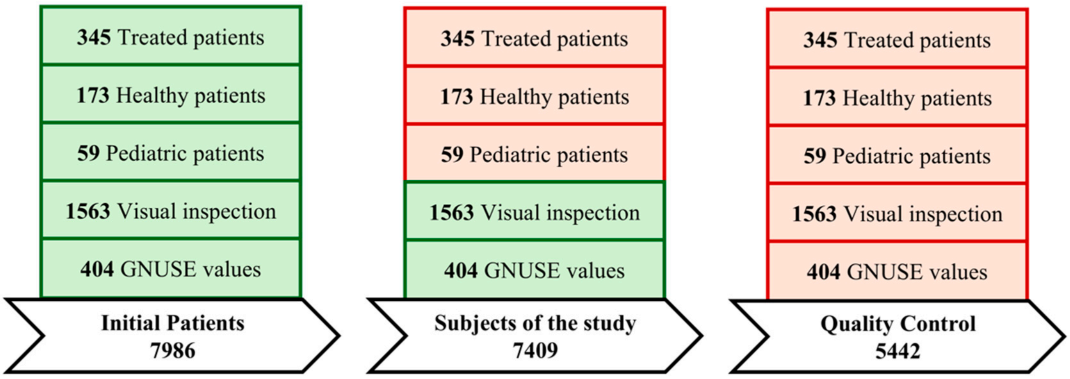

Supplementary Figure S4. *Flowchart of the sequential quality control and filtering actions performed for cleaning the original dataset.*

S7. Adjusting for batch effects

The heterogeneous origin of the available data challenge us to establish whether unwanted bias emerge in our final cohort. Both principal Components Analysis (PCA) and t-distributed stochastic neighbor embedding (t-SNE) on fRMA-processed data clearly hints at the presence of unwanted confusing factors.

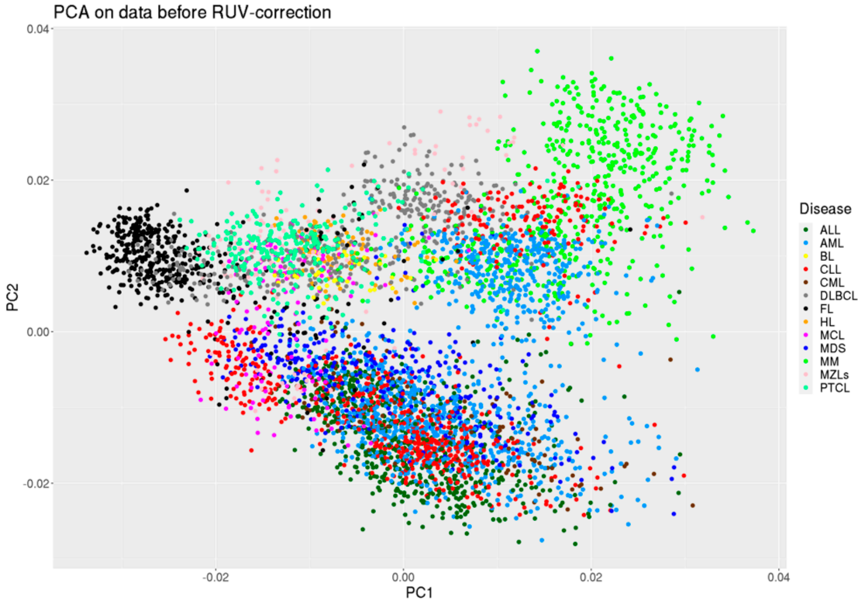

Supplementary Figure S4. *Overlook of the fRMA-processed data over the first two principal components. We notice several cases where subjects suffering the same HM split in well-distinct groups. This urges to account for batch-effects.*

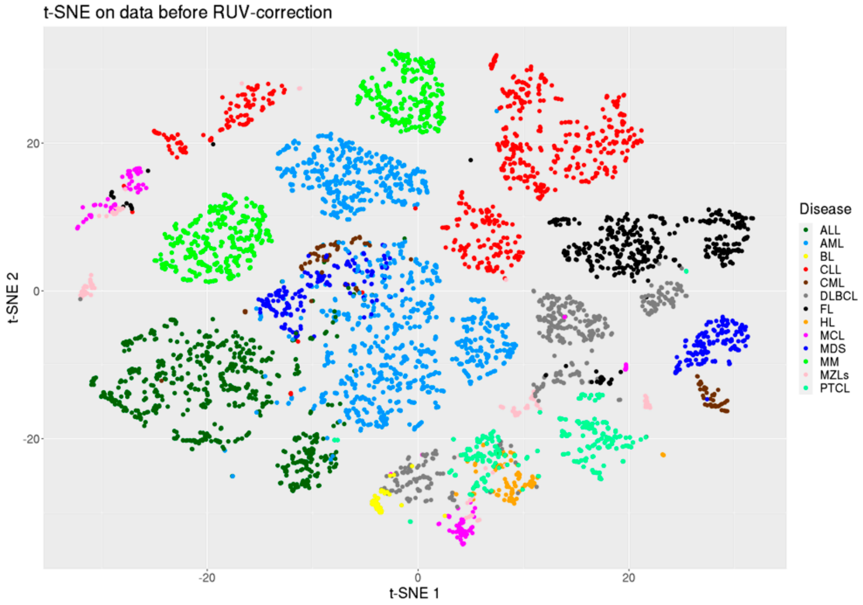

Supplementary Figure S5. *Distribution of latent-embedded subjects resulting from t-SNE on fRMA-processed data. We stress how most diseases organize in multiple disjunct groups. Unwanted confusing factor can reasonably explain this scenario, which is why we perform batch-correction.*

Our batch effect approach strongly relies on the wide-spread assumption expecting cancer to cause transcriptional changes only in a small number of genes. Given that, we can estimate unwanted signal over a subset of genes that we presume to unrelate to cancer biology, i.e., negative control genes. We adopt the Least Variant Set, or LVS, approach to estimate control genes. This approach seeks microarray probesets (associated with genes) playing no role with the

biological effect and whose variability depends exclusively on systematic variability. We run LVS on each dataset individually and we obtain 34 lists of negative control probesets. We choose to take the intersection of the lists to ultimately define the practical list of negative control probesets. Before moving forward with the actual batch-correction, we remove any potentially probeset that can be relevant for HMs. We end up with 9381 negative control probesets. Next, we perform Removal Unwanted Variation (RUV) endowed with the negative control probesets, which are later discarded in the downstream analyses. It is worthy to underline that we implement to the RUV procedure a term adjusting for HMs to ensure partial signal preservation. To optimize parameters for RUV we run a grid search over more than a thousand sets of parameters, and we select the best set with a metric based on the silhouette score. Our metric to select the optimal RUV parameters is a squared average of the silhouette scores (using Euclidean distance). In detail, (i) we compute the silhouette score for each GEO dataset on the first two principal components, and, since we intend to remove the systematic impact of the datasets, (ii) we calculate the squared average of the silhouette scores in order to (iii) choose the RUV parameters minimizing this metric. This is because we should ideally end up in a scenario where all GEO datasets are completely mixed and have no influence on how our data group together, which means that each silhouette score for a dataset is zero. At the end of our grid search we determine the following optimal parameters: 99 unwanted factors and Ridge penalty equal to one.

Once we complete correction with RUV, we translate from probesets to genes according to this formula:

$$y(g) = \sum_{p \in \rho(g)} w_p x_p$$

where $\rho(g)$ indicates the group of probesets associated with the g-th gene and $w_p = \frac{1}{n_p}$, with n_p being the number of genes covered by the p-th probeset. At this point, projections of the data along the first two components portrait compact groups associated with the HMs. Similarly, we achieve analogous outcomes with t-SNE.

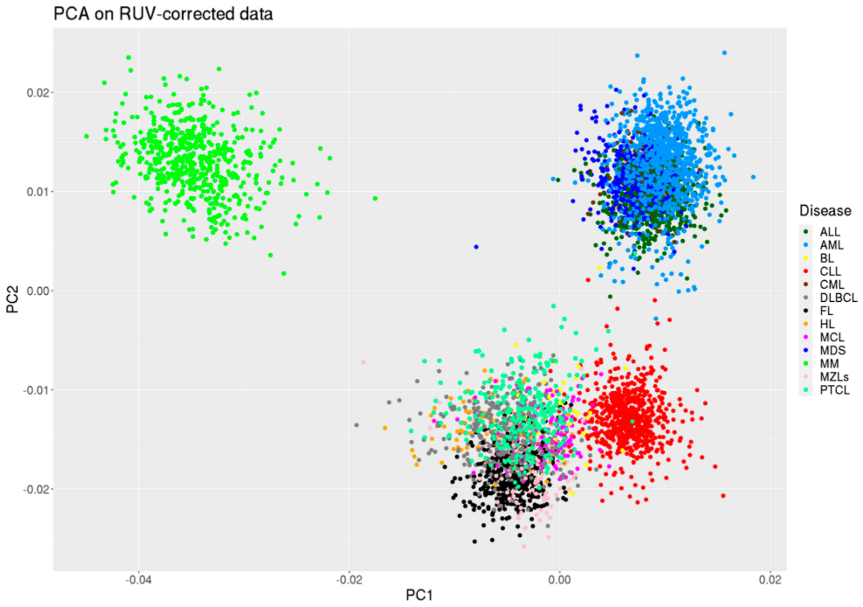

Supplementary Figure S6. *PCA projections after batch-correction with Removal of Unwanted Variation (RUV). Compact groups result for all HMs.*

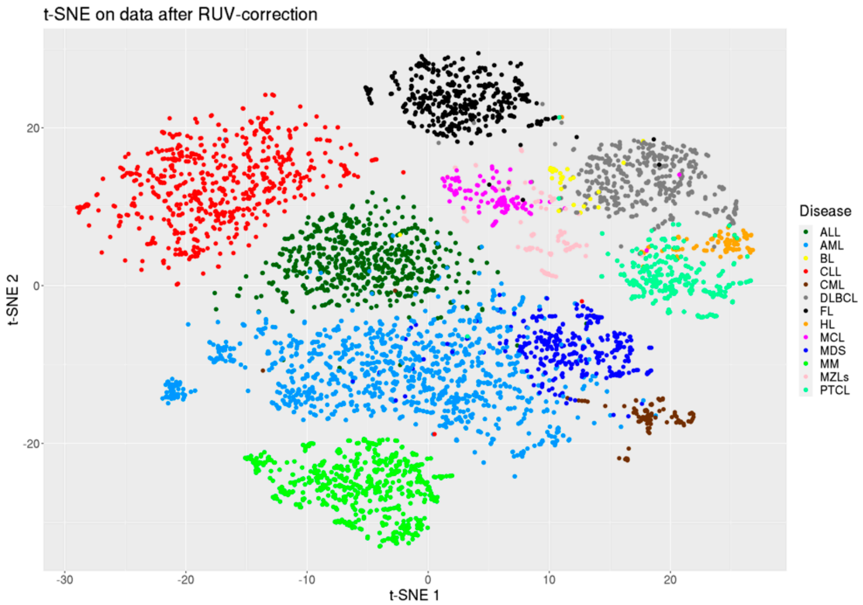

Supplementary Figure S7. *Organization of subjects according to t-SNE embedding of batch-corrected data. Each HM has subjects spreading consistently with very few exceptions.*

S7.1 Observing effect of batch correction per disease

We also control whether the systematic effect provided by the several GEO datasets continue to influence our data following the employment of RUV. Supplementary Figure S8 show the impact of the adjustment individually per disease. Each figure focuses on the PCA projections of the subjects affected by the same disease. In other words, to show the scenario before the correction for a single disease, we take the PCA result of Supplementary Figure S4 and we focus only on those subject affected by the disease. We do the same to display the scenario after the correction, in this case wrt Supplementary Figure S6.

We can appreciate qualitatively that in all disease the tendency to organize based on the GEO dataset is greatly reduced. This outcome, along with the overall PCA plot in Supplementary Figure S6, is a suggestion that, after the RUV correction, we determine a scenario where subjects with the same disease are consistent and both differences and similarities across diseases eventually can emerge.

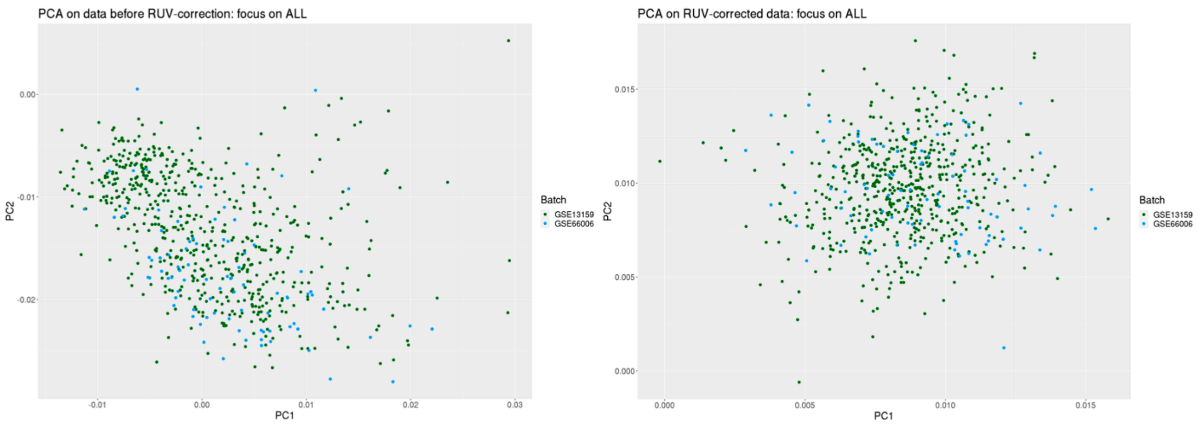

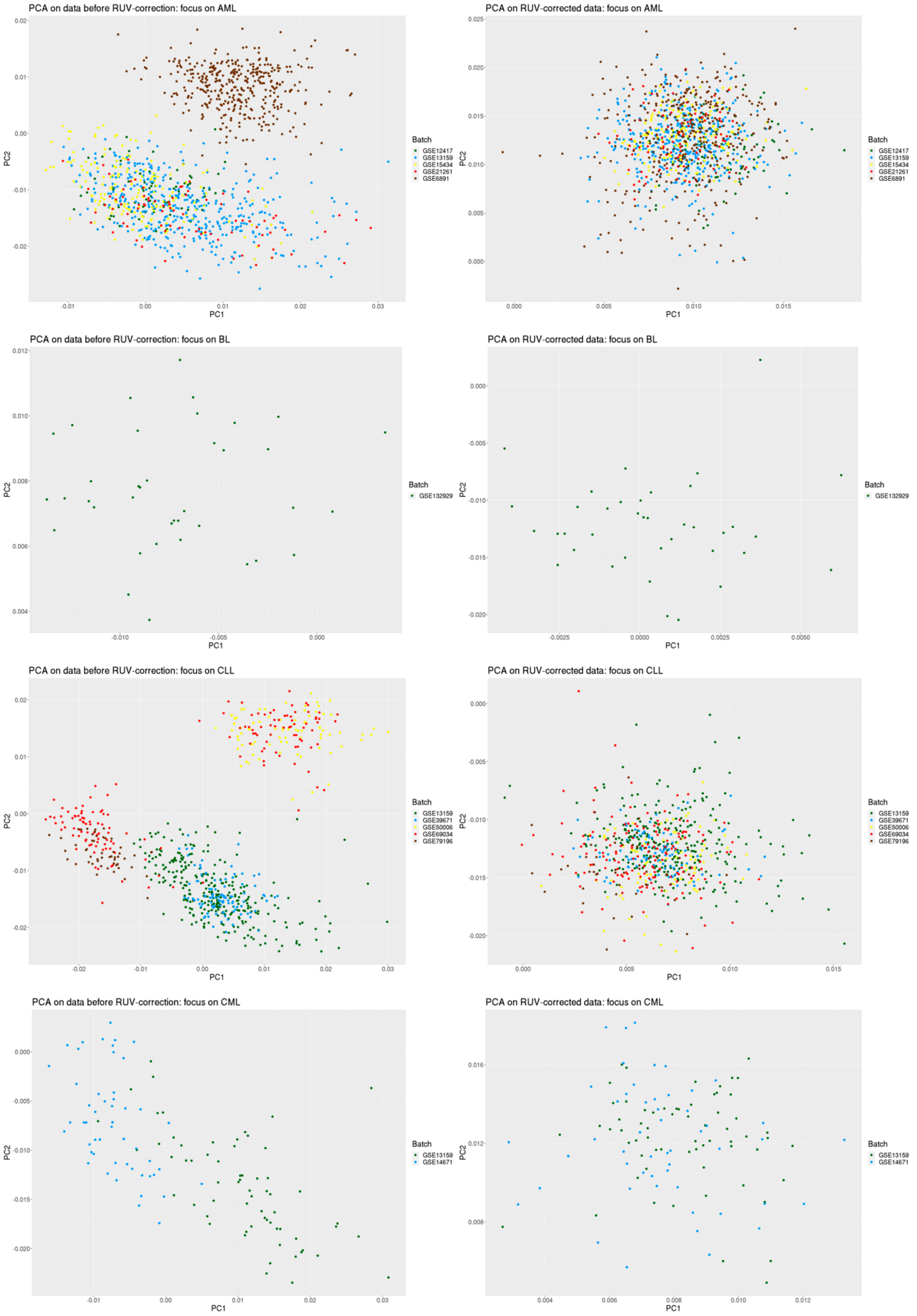
PCA on data before RUV-correction: focus on AML
PCA on RUV-corrected data: focus on AML
Batch
GSE12417
GSE13159
GSE15434
GSE21261
GSE6891
PCA on data before RUV-correction: focus on BL
PCA on RUV-corrected data: focus on BL
Batch
GSE132929
PCA on data before RUV-correction: focus on CLL
PCA on RUV-corrected data: focus on CLL
Batch
GSE13159
GSE39671
GSE50006
GSE69034
GSE79196
PCA on data before RUV-correction: focus on CML
PCA on RUV-corrected data: focus on CML
Batch
GSE13159
GSE14671
PC1
PC2

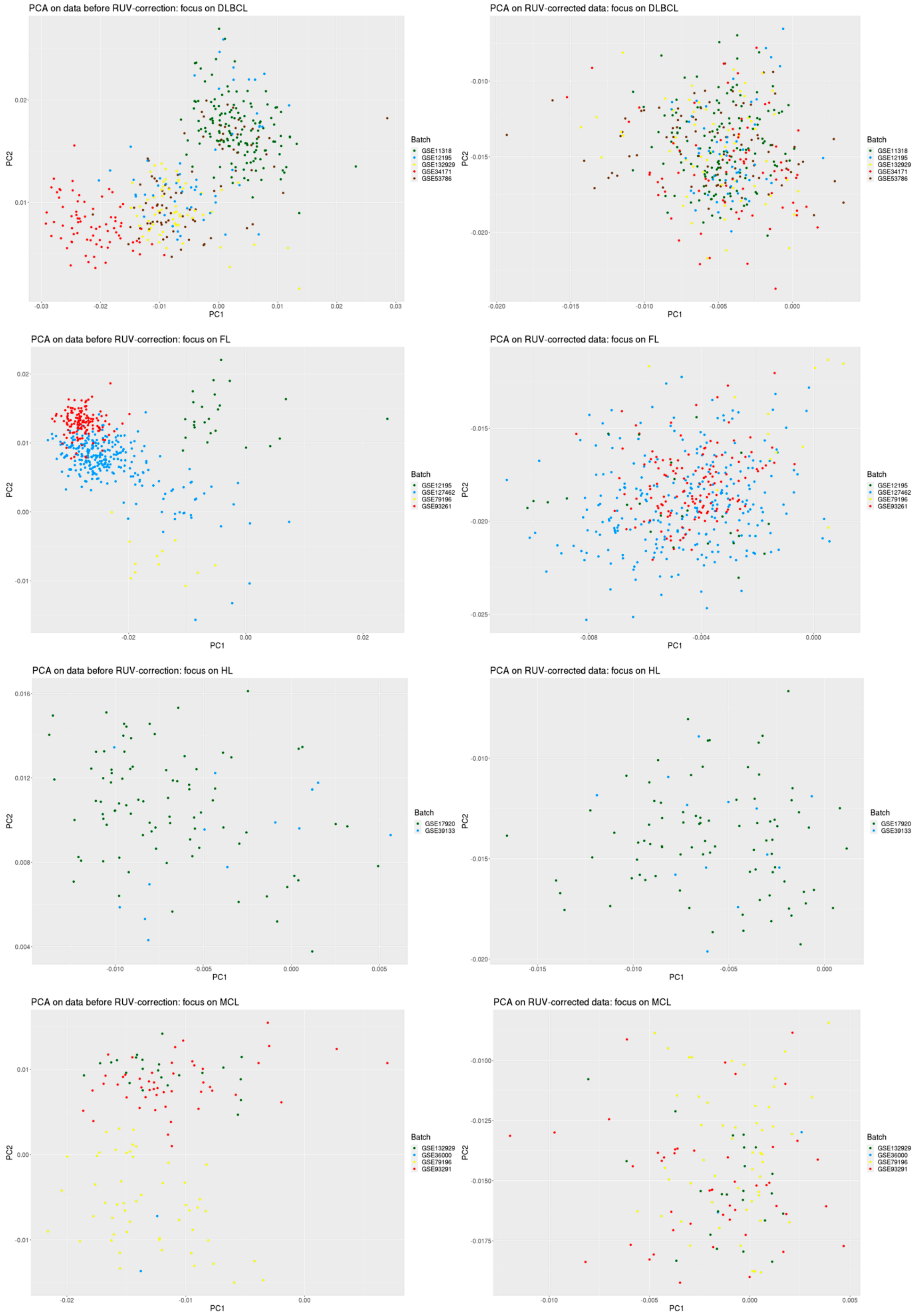

PCA on data before RUV-correction: focus on DLBCL
PCA on RUV-corrected data: focus on DLBCL
Batch
GSE11318
GSE12195
GSE132929
GSE34171
GSE53786
PCA on data before RUV-correction: focus on FL
PCA on RUV-corrected data: focus on FL
Batch
GSE12195
GSE127462
GSE79196
GSE93261
PCA on data before RUV-correction: focus on HL
PCA on RUV-corrected data: focus on HL
Batch
GSE17920
GSE39133
PCA on data before RUV-correction: focus on MCL
PCA on RUV-corrected data: focus on MCL
Batch
GSE132929
GSE36000
GSE79196
GSE93291
PC1
PC2

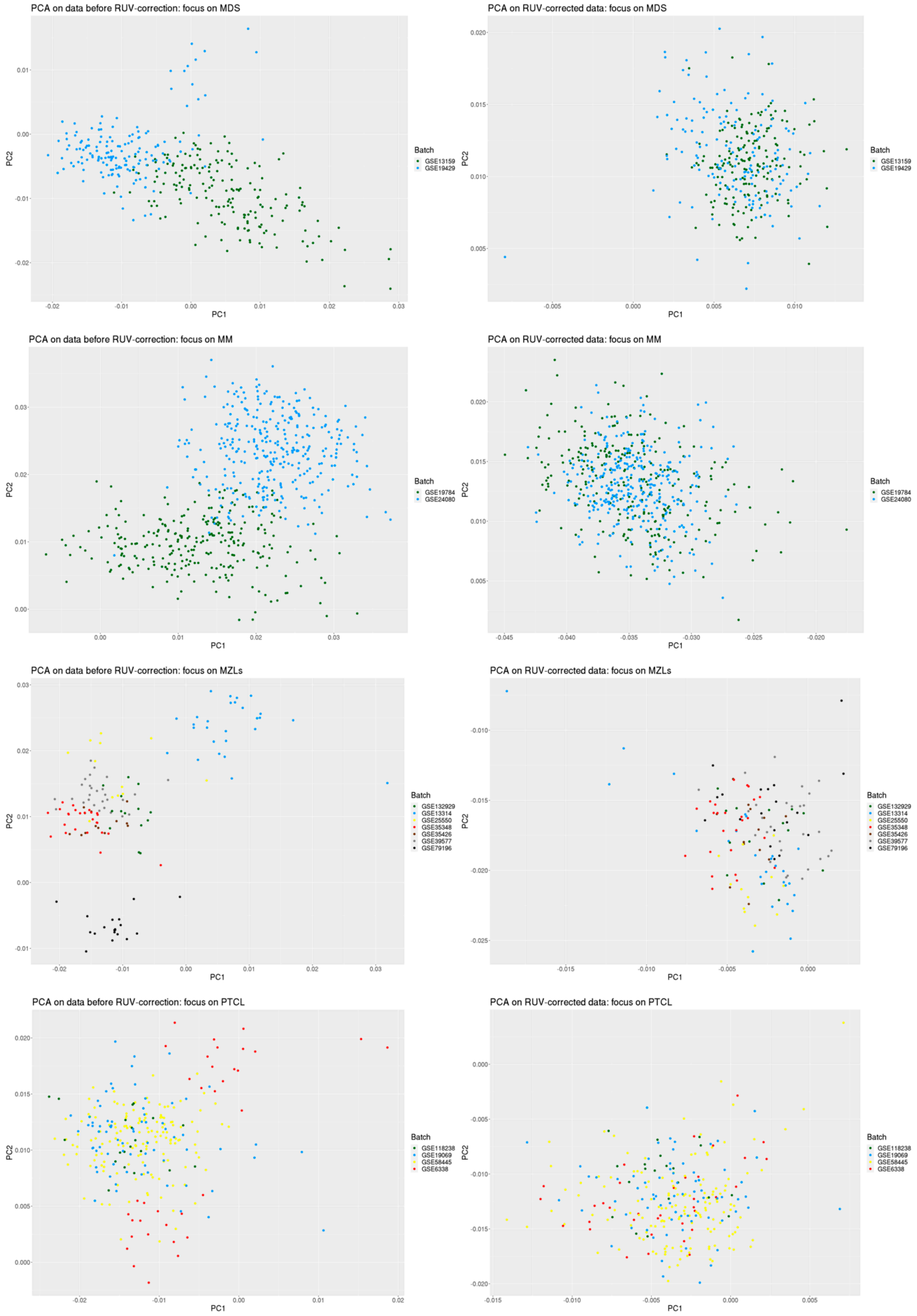
PCA on data before RUV-correction: focus on MDS
PC1
PC2
Batch
GSE13159
GSE19429
PCA on RUV-corrected data: focus on MDS
PC1
PC2
Batch
GSE13159
GSE19429
PCA on data before RUV-correction: focus on MM
PC1
PC2
Batch
GSE19784
GSE24080
PCA on RUV-corrected data: focus on MM
PC1
PC2
Batch
GSE19784
GSE24080
PCA on data before RUV-correction: focus on MZLs
PC1
PC2
Batch
GSE132929
GSE13314
GSE25550
GSE35348
GSE35426
GSE39577
GSE79196
PCA on RUV-corrected data: focus on MZLs
PC1
PC2
Batch
GSE132929
GSE13314
GSE25550
GSE35348
GSE35426
GSE39577
GSE79196
PCA on data before RUV-correction: focus on PTCL
PC1
PC2
Batch
GSE118238
GSE19069
GSE58445
GSE6338
PCA on RUV-corrected data: focus on PTCL
PC1
PC2
Batch
GSE118238
GSE19069
GSE58445
GSE6338

Supplementary Figure S8. *Effect of RUV correction on each disease type. Colors across the subfigures indicate the original GEO dataset (i.e., causing the batch effect) for each subject. The reduction of batch effect can be observed in all diseases, suggesting that after the RUV correction the subjects affected by the same disease are more consistent with each other.*

S8. Comment on distance metric for expression

We define a distance metric, over the specificity scores of expression levels, that is needed to perform hierarchical clustering. Such score has the following form:

$$s_j^{dis} = \frac{med\left(e_j^{dis}\right) - med\left(e_j\right)}{IQR\left(e_j\right)}$$

Then, given diseases A and B, the Euclidean distances of their scores turns out to be:

$$\left|\left|s^A, s^B\right|\right| = \sqrt{\sum_{j}^{N\ genes} \left(\frac{med\left(e_j^A\right) - med\left(e_j^B\right)}{IQR\left(e_j\right)}\right)^2}$$

That is, the expression-based distance between two diseases is obtained by comparing the median expressions characteristic to the two diseases and weighting genes such to give more importance to the less variabile ones. Due to this implementation of feature importance, the so-called *curse of dimensionality*, which would make the Euclidean distance meaningless in high-dimensional spaces, is avoided.

S9. Distribution of the specificity score of expression level

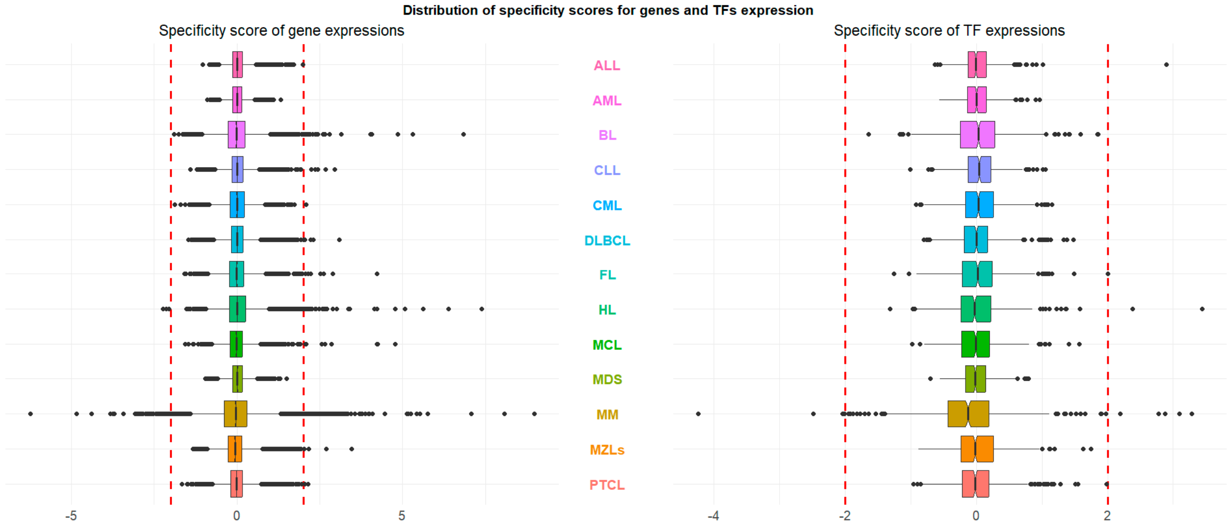

Supplementary Figure S9. *Distribution of the specificity score of gene (left) and TF (right) expression, for each disease. Outliers are defined by a score value greater than 2.*

S10. Complete table of HM-specific genes

**Cluster of HMs**	**Specific genes**
BL	BEST3, BGN, BMP7, CADM1, CADPS, COL4A2, DDX11-AS1, FAM167A, GJC1, GPR116, IGF2BP1, LAMB1, LZTS1, MMRN2, NAPSA, PDZD2, PNMAL1, POSTN, PPM1E, RASSF6, S1PR2, TERT, THBS2, UCHL1, UGT8, ZNF385B
BL-DLBCL	AICDA
BL-DLBCL-FL	RGS13
BL-DLBCL-HL	RARRES1
CLL	ABCA6, CLNK, PHEX
CLL-MCL	LOC101929777, ROR1
CML	SFRP1
DLBCL	NUPR1
DLBCL-FL	ZBED2
FL	EML6, GPR82, HTR3A, IGH, LOC100506457, SYBU
HL	ALOX15, ANKRD29, BCL2L14, CCDC102B, CCL11, CCL13, CCL17, CCL22, CCL24, CCL26, CCL8, CYP4Z1, DHRS2, EGFL6, FMO2, HSD11B1, IL13, IL13RA2, LINC01094, LOC101930114, NTS, PVALB, RCAN2, RP11-664D1.1, SFRP2, TENM2, TFPI2, TNIP3
HL-MZLs	ADH1B
HL-MZLs-PTCL	FDCSP
MCL	ADAMTS6, CCND1, CHL1, LILRA4, PPP1R17, SOX11
MM	ABCB9, ACY1, AGA, ALDH1L2, ALG1, ALG14, ALG2, ALG3, ALG5, ALG8, ALG9, AMPD1, APOA1BP, ARL1, ASS1, ATG4A, ATP8B2, ATRAID, AVEN, B4GALT3, B9D1, BEX5, BMI1, BMP6, BMP8B, BSCL2, BTD, C11orf1, C15orf65, C19orf10, C19orf70, C1GALT1C1, CADPS2, CALU, CBS, CBWD1, CBWD1, CBWD1, CCPG1, CERCAM, CHAC2, CHID1, CHPF, CISD2, CLCC1, CLPTM1L, CNPY2, COMMD3, COQ4, COX18, CRELD2, CTSO, CUTA, CYB561, DDOST, DDRGK1, DEPTOR, DERL1, DERL3, DKK1, DNAJB11, DNAJB9, DNAJC1, DPAGT1, DPM3, DUSP5, DYX1C1-CCPG1, EDEM2, EDEM3, EMC7, ERGIC2, ERLEC1, ERN1, EYA2, FAM149A, FAM174A, FICD, FITM2, FKBP11, FRZB, FUCA2, GARS, GAS6, GFPT1, GLT8D1, GOLT1B, GORASP2, GPR89A, GPR89B, GPRC5D, HID1, HM13,

	HRASLS2, HSD17B8, HSPA13, IFNAR1, IGF1, IL5RA, IMPAD1, INHBE, INPP4A, ITGA8, KCNN3, KDELR1, KDELR2, KDELR3, KIAA1244, LAMP3, LAMP5, LINC00582, LMAN2, LMF1, LOC101928061, LRRN1, LYSMD3, MAGED2, MAGT1, MAN2A1, MANEA, MANSC1, MCC, MCEE, MEI1, MESDC2, MGAT2, MIR3180-1, MIR7110, MKX, MOXD1, MTDH, MYEOV, NDNF, NEU1, OGFOD3, OSTC, P4HTM, PAIP2B, PARM1, PCYOX1, PDIA4, PDIA6, PERP, PGRMC2, PIGP, PMEPA1, PMVK, PPIB, PRADC1, PRDX4, PREB, PRELID1, PRICKLE2, PSAT1, RABAC1, RNF148, RPN1, SAR1B, SCAMP5, SDC1, SDF2L1, SEC11C, SEC61A1, SEL1L, SIGMAR1, SIL1, SLAMF7, SLC1A4, SLC1A5, SLC35B1, SLC35B3, SLC38A10, SLC52A2, SMOC1, SPAG4, SPATS2, SPCS1, SPCS2, SPCS2, SPCS3, SRPRB, SSPN, SSR1, SSR2, SSR3, SSR4, SSSCA1, ST3GAL6, SURF4, TAPBPL, TBC1D30, TBL2, TCTN3, TECR, TJP1, TM9SF4, TMCO1, TMEM106B, TMEM192, TMEM205, TMEM208, TMEM258, TMEM39A, TMEM45A, TMEM5, TRAM1, TTLL7, TXNDC11, TXNDC15, UAP1, UBA5, UBE2QL1, UGT2B17, USO1, VIMP, VKORC1, WFS1, WIPI1, WNT5A, YIF1A, YIPF2
MZLs	CYR61, FCRL4, SFTPC
PTCL	CCL21

Supplementary Table S5. *Report of clusters defined by specific genes. We list all specific genes of a disease cluster, indicating in red the ones associated or potentially associated to the HMs.*

S11. Complete table of HM clusters defined by commonly enriched biological pathways

In Supplementary Table S5 below, each cluster of HMs is associated with the biological functions commonly enriched by that set of diseases. GSEA analysis also provides the list of genes which mostly determined the enrichment, called driving genes. The number of driving genes common to all the diseases of each cluster is reported next to each pathway. The complete list of such genes (for each pathway), along with the specification of the ones which can be targeted by approved drugs, is available in Table S1.

Cluster of HMs	Enriched KEGG pathways
MM	KEGG_REGULATION_OF_ACTIN_CYTOSKELETON (74), KEGG_FC_GAMMA_R_MEDIATED_PHAGOCYTOSIS (47), KEGG_T_CELL_RECEPTOR_SIGNALING_PATHWAY (45), KEGG_N_GLYCAN_BIOSYNTHESIS (31), KEGG_FC_EPSILON_RI_SIGNALING_PATHWAY (30),

	KEGG_NOD_LIKE_RECEPTOR_SIGNALING_PATHWAY (28), KEGG_TYPE_II_DIABETES_MELLITUS (22), KEGG_OTHER_GLYCAN_DEGRADATION (8)
CML	KEGG_ENDOCYTOSIS (58), KEGG_INSULIN_SIGNALING_PATHWAY (44), KEGG_NEUROTROPHIN_SIGNALING_PATHWAY (43), KEGG_ERBB_SIGNALING_PATHWAY (24), KEGG_ENDOMETRIAL_CANCER (20), KEGG_PENTOSE_PHOSPHATE_PATHWAY (14), KEGG_THYROID_CANCER (12)
CLL	KEGG_PURINE_METABOLISM (49), KEGG_B_CELL_RECEPTOR_SIGNALING_PATHWAY (37), KEGG_CYSTEINE_AND_METHIONINE_METABOLISM (18), KEGG_PRION_DISEASES (11), KEGG_PENTOSE_AND_GLUCURONATE_INTERCONVERSIONS (6)
AML-CLL-CML-DLBCL-FL-HL-MCL-MDS-MM-MZLs-PTCL	KEGG_CELL_ADHESION_MOLECULES_CAMS (5), KEGG_ALLOGRAFT_REJECTION (4), KEGG_AUTOIMMUNE_THYROID_DISEASE (4)
ALL	KEGG_LYSOSOME (41), KEGG_DNA_REPLICATION (21), KEGG_SPHINGOLIPID_METABOLISM (14)
MM-MZLs	KEGG_OXIDATIVE_PHOSPHORYLATION (35), KEGG_PARKINSONS_DISEASE (31)
AML-CML-MDS	KEGG_CHRONIC_MYELOID_LEUKEMIA (18), KEGG_ACUTE_MYELOID_LEUKEMIA (16)
AML-CLL-CML-FL-HL-MDS-MM-MZLs-PTCL	KEGG_GRAFT_VERSUS_HOST_DISEASE (5), KEGG_TYPE_I_DIABETES_MELLITUS (5)
MZLs	KEGG_ALZHEIMERS_DISEASE (69)
MDS-MZLs	KEGG_HUNTINGTONS_DISEASE (56)
MDS-MM	KEGG_HEMATOPOIETIC_CELL_LINEAGE (17)
FL-MM-MZLs	KEGG_PROTEIN_EXPORT (9)
DLBCL-MM	KEGG_LEUKOCYTE_TRANSENDOTHELIAL_MIGRATION (12)
CML-MM	KEGG_PRIMARY_IMMUNODEFICIENCY (7)
CLL-MM	KEGG_NATURAL_KILLER_CELL_MEDIATED_CYTOTOXICITY (22)
CLL-MDS	KEGG_PEROXISOME (17)
CLL-FL-MM	KEGG_LEISHMANIA_INFECTION (11)
CLL-CML-FL-HL-MCL-MDS-MM-PTCL	KEGG_ASTHMA (6)
AML-FL	KEGG_PROGESTERONE_MEDIATED_OOCYTE_MATURATION (23)
AML-DLBCL-FL-HL-MDS-MM-MZLs-PTCL	KEGG_SYSTEMIC_LUPUS_ERYTHEMATOSUS (8)

AML-CML-DLBCL-FL-HL-MDS-MM-PTCL	KEGG_ANTIGEN_PROCESSING_AND_PRESENTATION (5)
AML-CML	KEGG_COLORECTAL_CANCER (19)
AML-CLL-CML-DLBCL-FL-MCL-MDS-MM-MZLs-PTCL	KEGG_VIRAL_MYOCARDITIS (5)
AML-CLL-CML-DLBCL-FL-MCL-MDS-MM-MZLs	KEGG_INTESTINAL_IMMUNE_NETWORK_FOR_IGA_PRODUCTION (4)
ALL-MZLs-PTCL	KEGG_RIBOSOME (39)
ALL-DLBCL-FL-HL-MCL-MM-MZLs-PTCL	KEGG_CHEMOKINE_SIGNALING_PATHWAY (8)
ALL-DLBCL	KEGG_ETHER_LIPID_METABOLISM (6)
ALL-AML-MDS-MM-MZLs	KEGG_SPLICEOSOME (30)
ALL-AML-FL-MDS-MZLs	KEGG_CELL_CYCLE (21)
ALL-AML-DLBCL-FL-HL-MCL-MDS-MM-MZLs-PTCL	KEGG_CYTOKINE_CYTOKINE_RECEPTOR_INTERACTION (9)
ALL-AML-BL-DLBCL-MCL-MDS-MZLs-PTCL	KEGG_FOCAL_ADHESION (22)
ALL-AML-BL-DLBCL-FL-HL-MCL-MDS-MZLs-PTCL	KEGG_COMPLEMENT_AND_COAGULATION_CASCADES (12)
ALL-AML-BL-CML-DLBCL-HL-MCL-MDS-MZLs-PTCL	KEGG_ECM_RECEPTOR_INTERACTION (12)

Supplementary Table S6. *Clusters of HMs based on commonly enriched biological function. Next to each pathway we report the number of driving genes shared by all the diseases within a cluster. Driving genes stand for the genes found by GSEA that mostly influenced the enrichment of a pathway.*

S12. Initialization of PANDA algorithm

Unlike some other network approaches, PANDA does not directly incorporate co-expression information between regulators and targets. Instead, the edges in PANDA-predicted networks reflect the overall consistency between a TF's canonical regulatory profile and its target genes' co-expression patterns. The weight of each TF-to-gene regulation is the similarity score used by PANDA, which adapts the Tanimoto similarity to be symmetric around 0 and to range from –K to +K rather than from –1 to +1, where K is a constant dependent on the size of the network. The units of such score are z-score units, therefore one can interpret in such a way the type of regulation associating any TF to any gene: large positive values mean strong positive regulation, while large negative values mean strong inhibition of the expression.

To set up PANDA, the position weight matrices (PWSs) of 644 transcription factors (TFs) were downloaded from the CIS-BP database [1] and the promoter regions of genes were downloaded from the most recent human genome release provided by UCSC [2]. Next, each TF was scanned along every region by the FIMO tool from the MEME suite [3] to estimate the probability of a motif in the promoters for a TF. These probabilities were then thresholded using value 10−5 to yield the initial agnostic binary regulatory network. Moreover, the TFs were mapped onto the STRING Protein Protein Interaction network (PPI) [4] to generate the initial TF-to-TF network. Such network was disease-agnostic. The initial network feeding PANDA with diverse biological input was the gene-to-gene network, that is approximated by the gene correlation matrix of each disease. Given these three networks, the PANDA algorithm was able to find an agreement for them. To be noted, negative control probesets were converted to negative control genes (4262) and were removed prior to PANDA. Also, genes and TFs that did not appear initially in both expression data and regulatory

network were excluded. Noteworthy, we follow the work Sonawane et al. [5] to select the 644 TFs for PANDA. Eventually, in our analyses we only consider 609 TFs, those with available expression data.

S13. Metric for regulation-based distance

The score for regulation specificity is defined as

$$s_{ij}^{dis} = \frac{e_{ij}^{dis} - med(e_{ij})}{IQR(e_{ij})}$$

Where e_{ij}^{dis} are the edges of the regulatory networks. By comparing the corresponding edges of two disease-specific regulatory networks, *A* and *B*, a Euclidean distance can be computed as

$$||s^A, s^B|| = \sqrt{\sum_{i}^{N\,TFs} \sum_{j}^{N\,genes} \left(s_{ij}^A - s_{ij}^B\right)^2}$$

from which pairwise distances between diseases can be defined. Using these regulation-based distances, hierarchical clustering has been performed via the dynamic tree cut of the dendrogram obtained with the complete linkage method.

S14. Further details on specific TF-gene regulations of HMs

HMs distribute heterogeneously on extremely large TF-gene regulations. We find a huge number of TF-gene regulations (n=429316, i.e., almost 3.25%) specific to at least one HM and, accordingly, combinations of HMs sharing the same specificities are extremely numerous. In this scenario (Supplementary Figure S10), FL stands out with the highest number of specific TF-gene regulations (n=179822, i.e., roughly 37.8% of the total), followed by BL (~12.9%) and HL (~10.5%). Around 80% of specificities characterize a single HM. 16% of TF-gene specificities are shared by couples of HMs, less than 3% by triplets of HMs. Supplementary Table S6 reports the couples of HMs along with the number of specific TF-gene regulations they share. Supplementary Figure S11 shows the couples associated by the highest number of TF-gene specificities. The couples BL-MCL and FL-PTCL are associated by slightly more than 5% of the specificities found overall, followed by the couples CML-Fl and CML-HL which around 4.5% of all the TF-gene specificities.

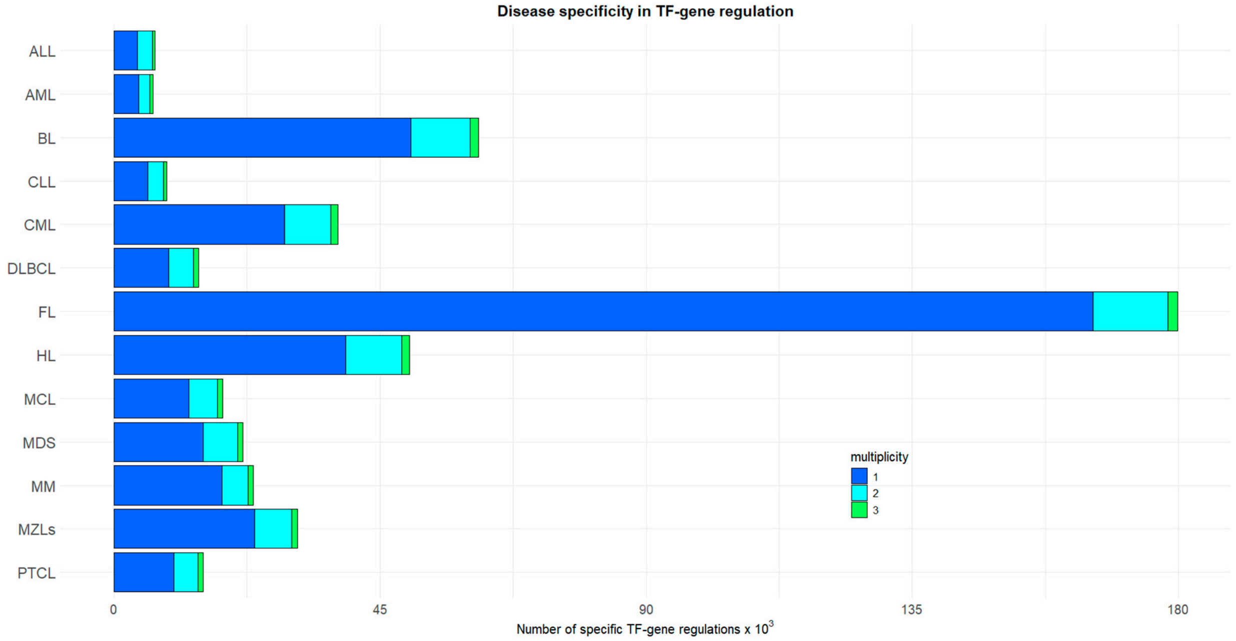

Supplementary Figure S10. *Number and multiplicity of disease-specific TF-gene regulation. The multiplicity of a regulation indicates the number of HMs for which such network weight is significatively different from the median value.*

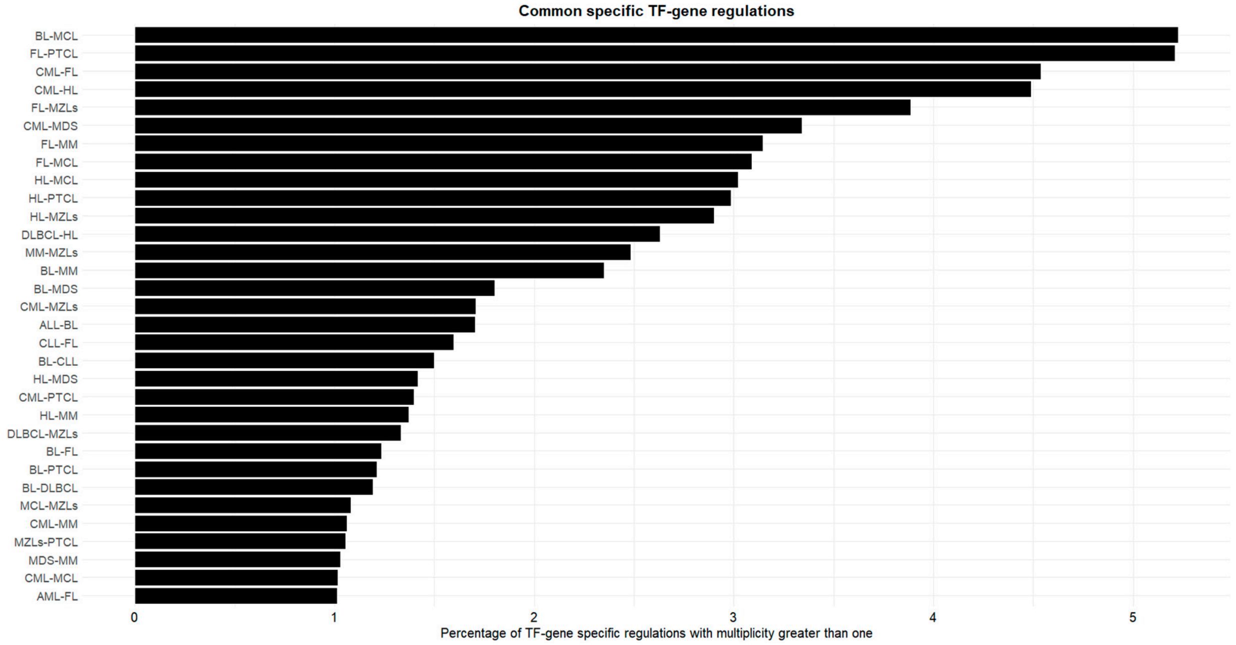

Supplementary Figure S11. *Distribution over disease couples of specific TF-gene regulations common to multiple diseases. The couples sharing more than 1% of the total specific TF-gene regulations with multiplicity greater than one are reported.*

S15. Cluster of HMs based on regulatory networks

Performing GSEA on each TF per HM, we keep exclusively significantly enriched pathways (adjusted p-value<0.05). This leaves us many options to explore. In this work, we are mainly interested in clusters of HMs that enrich on the same KEGG pathway but (i) through the same TF and (ii) sharing the genes that drove the enrichment (also known as leading genes). Therefore, we are not just looking at associations between groups of diseases and biological functions,

because we also want to know which TF causes such an enrichment and through which genes. In this way we might be able to speculate that multiple HMs pivot on a specific TF to regulate a set of genes, ultimately damaging the functionality of a biological function. Our choice was to report in the manuscript only those HMs clusters whose common TF is also drug targeted. For the sake of clarity, we mention that after GSEA we obtain 90 HMs clusters. Upon considering identical TF and the presence of common driving genes, only 40 clusters remain. Multiple clusters carry many combinations of TF and pathway. Now, if we filter based on the drug-targetable TFs, we are left with Table 2 of the main text. If we do not, Table S2 results.

[1] M. T. Weirauch *et al.*, "Determination and inference of eukaryotic transcription factor sequence specificity.," *Cell*, vol. 158, no. 6, pp. 1431–1443, Sep. 2014, doi: 10.1016/J.CELL.2014.08.009/ATTACHMENT/98B80ADD-104E-40CD-B7E6-4E71B364ADFC/MMC5.XLSX.

[2] W. James Kent *et al.*, "The Human Genome Browser at UCSC," *Genome Res*, vol. 12, no. 6, pp. 996–1006, Jun. 2002, doi: 10.1101/GR.229102.

[3] C. E. Grant, T. L. Bailey, and W. S. Noble, "FIMO: scanning for occurrences of a given motif," *Bioinformatics*, vol. 27, no. 7, pp. 1017–1018, Apr. 2011, doi: 10.1093/BIOINFORMATICS/BTR064.

[4] D. Szklarczyk *et al.*, "The STRING database in 2021: customizable protein–protein networks, and functional characterization of user-uploaded gene/measurement sets," *Nucleic Acids Res*, vol. 49, no. D1, p. D605, Jan. 2021, doi: 10.1093/NAR/GKAA1074.

[5] A. R. Sonawane *et al.*, "Understanding Tissue-Specific Gene Regulation," *Cell Rep*, vol. 21, no. 4, pp. 1077–1088, Oct. 2017, doi: 10.1016/J.CELREP.2017.10.001/ATTACHMENT/8E865D81-D76B-4389-9C46-0C7F5B683D46/MMC9.PDF.
